# Disruption of cellular immune response among male rotating night shift workers in Spain– The HORMONIT study

**DOI:** 10.3389/fimmu.2022.776917

**Published:** 2022-09-02

**Authors:** Barbara N. Harding, Ruth Aguilar, Ana Espinosa, Gemma Castaño-Vinyals, Kyriaki Papantoniou, José Maria Navarrete, Patricia Such Faro, Antonio Torrejón, Carlota Dobaño, Gemma Moncunill, Manolis Kogevinas

**Affiliations:** ^1^Department of Non-Communicable Diseases and Environment, Barcelona Institue of Global Health (ISGlobal), Barcelona, Spain; ^2^Universitat Pompeu Fabra (UPF), Barcelona, Spain; ^3^CIBER Epidemiología y Salud Publica (CIBERESP), Madrid, Spain; ^4^Barcelona Institue of Global Health (ISGlobal), Hospital Clínic, Universitat de Barcelona, Barcelona, Spain; ^5^IMIM (Hospital del Mar Medical Research Institute), Barcelona, Spain; ^6^Department of Epidemiology, Center for Public Health, Medical University of Vienna, Vienna, Austria; ^7^Health, Safety and Emergencies of SEAT, CUPRA and the Volkswagen Group Companies in Spain, Barcelona, Spain; ^8^CIBER de Enfermedades Infecciosas (CIBERINFEC), Barcelona, Spain

**Keywords:** cytokines, chemokines, growth factors, immune response, night shift, rotating night shift workers

## Abstract

**Introduction:**

Preliminary studies suggest that night shift work is associated with a desynchronization of rhythmic immune markers, possibly explaining the increased risk of infection, cardiometabolic disorders, and cancer in shift workers.

**Methods:**

This study included 51 male rotating shift workers from a car industry in Barcelona, Spain, sampled twice toward the end of a 3-week night shift (22:00-06:00 h) and a 3-week day shift (06:00-14:00 h) rotation. We collected four blood samples per worker, at the start and end of each shift. We measured 27 cytokines, chemokines and growth factors in plasma samples by luminex using the Cytokine Human Magnetic 30-Plex Panel LHC6003M and applied linear mixed models to examine within-person associations between shift work and analytes’ concentrations, comparing samples taken at 06:00 h on a day and night shift. We also conducted a factor analysis using analyte concentrations from all 4 time points for each individual to identify common factors and determine if these factors were altered by shift work.

**Results:**

We observed lower levels of 15 analytes in the night shift compared to the day shift including cytokines (pro-inflammatory TNF-α, IL-2R; anti-inflammatory IL1-RA; Th1 IL-2, Th2 IL-4 and Th17 Il-17), chemokines (IP-10, MIP-1α, MIP-1β, RANTES) and growth factors (EGF, G-CSF, HGF, VEGF, FGF). In a factor analysis, three factors were identified. The main factor (Factor 1), explaining 57% of the variance and including IL-1β, IL-12, IL-15, MIP-1α, MIP-1β, EGF and FGF; and another factor (Factor 3) explaining 10% of the variance and including the Th1 cytokine IL-12, were inversely associated with the night shift (coefficient: -0.17, 95%CI -0.32 to -0.01 and coefficient: -0.22, 95%CI -0.38, -0.06, for Factors 1 and 3, respectively). Our results indicate that night shift disrupts the levels of several immune markers, which could contribute to the increased risk of infections and cancer reported in night shift workers.

**Conclusion:**

Night shift is associated with disruption of multiple immune response pathways.

## Introduction

Circadian rhythms originate from an organisms’ intrinsic ability to align countless physiologic processes to the environmental 24 h light and dark cycle (1, 2). Most immune cells express circadian clock genes (3, 4), which have profound impacts on cellular abundance and rate of DNA synthesis (5, 6). Percentages of human blood mononuclear cells, T cells, and B cells show a clear peak at night, followed by a depression in levels in the morning. Conversely, antibody-dependent cytotoxicity of lymphocytes peak in the day and decline at night (7). Daily circadian rhythms also affect cellular function by altering the synthesis and release of cytokines, chemokines and growth factors, well-known mediators of immune responses to infection (1). Circadian rhythms have been reported for cytokine levels in the blood (8–11), and following ex vivo stimulation with various stimuli (3, 12–15). Circadian rhythms have been shown to have a critical role in immune system homeostasis regulating the innate and adaptive immune systems in rodents and humans (16).

In conjunction with circadian rhythms, sleep is known to regulate immune functions (17, 18). However, it is difficult to distinguish the respective influence of these two regulatory systems given their interconnectedness (19). Investigations of the normal sleep–wake cycle show that the production of pro-inflammatory cytokines exhibit peaks during early nocturnal sleep whereas the anti-inflammatory cytokine activity peaks during daytime wakefulness (19). As a result, mistimed sleep, as occurs after night shift work, and the consequent alterations of circadian rhythms are suggested to profoundly alter the rhythmic profiles of immunocytes in the blood and their production of various cytokines, negatively affecting immune responses to disease (1).

Immunology data suggest that night shift work is associated with a desynchronization of rhythmic immune parameters (20–24), which might contribute to the increased risk for infection (25, 26), autoimmune diseases (25), cardiovascular and metabolic disorders (20, 27, 28), and cancer reported in shift workers (29). There are many cardiovascular disease conditions in which a circadian influence on inflammation has been suggested to play a pathophysiological role: myocardial infarction (30), atherosclerosis (31), vascular biology (32), stroke (33), and chronic heart failure (34). A recent study in a mouse model shows that circadian disruption significantly increases cancer-cell dissemination and lung metastasis in breast cancer. It also enhances the stemness and tumor-initiating potential of cells and creates an immunosuppressive shift in the tumor cytokine/chemokine microenvironment (35). Some studies in humans have also shown an association of night shift work with a higher frequency of infectious disease, also suggestive of immunosuppression (36, 37).

However, results from studies examining levels of immune markers and inflammation have produced conflicting results and have been limited by small sample sizes and methodologic shortcomings including the lack of adjustment for potential confounding factors (38). Additional studies that better characterize immune disruption through a wide range of biomarkers and repeated measurements among shift workers are needed to characterize the effects of such disruption on the functions of the innate and adaptive immune systems.

We undertook the present study to examine how night shift work alters cellular immune markers within a population of male rotating night shift workers in Spain. Specifically, we examined within participant differences in the levels of cytokines, chemokines and growth factors after exposure to several days of night work compared to levels after several days of day work. We also evaluated whether age or chronotype, a characteristic correlating with preference for morning or evening activity (39), modified changes in levels of immune markers.

## Materials and methods

### Study population

**Figure 1:**
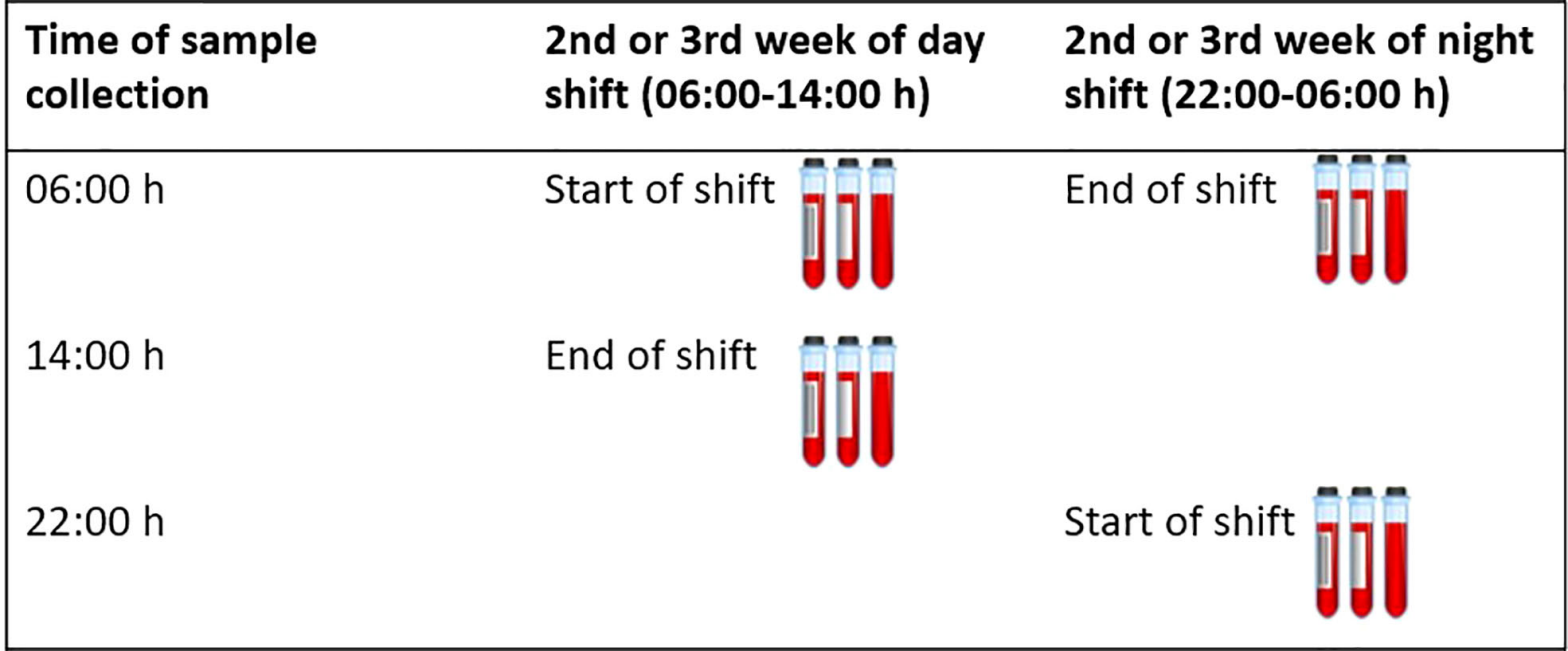
Timing of blood collection. This figure shows when blood samples were collected for each participant. A total of 4 samples were collected. Two samples were collected on a day shift during the 2^nd^ or 3^rd^ week of the rotation (one sample at the start and one at the end of the shift) and two samples were collected on a night shift during the 2^nd^ or 3^rd^ week of the rotation (one sample at the start and one at the end of the shift).

This study included male rotating shift workers between the ages of 18-65 from the HORMONIT study (Molecular Epidemiological Study on Hormonal Changes Associated with Circadian Disruption in Night Shift Workers) (40) who were sampled from a car industry in Barcelona, Spain. Workers were assigned to a given shift for 3-week stretches before switching to a different shift pattern. Workers rotated backwards through shifts: night shift (22:00-06:00 h), evening shift (14:00-22:00 h), and morning shift (06:00-14:00 h). Workers worked five-day work weeks (Monday-Friday) with two days off (Saturday and Sunday). We took 4 blood samples per worker. We collected two samples on a day shift during the 2^nd^ or 3^rd^ week of the rotation (one sample at the start and one at the end of the shift) and two samples on a night shift during the 2^nd^ or 3^rd^ week of the rotation (one sample at the start and one at the end of the shift), and compared cytokine levels between sampling time-points and working shifts within the same participant. We did not collect samples from participants on Mondays to avoid some potential influence of the weekend on cytokine levels. The sample taken at the start of a day shift was taken at 06:00 h which corresponded to the same time a sample was taken at the end of the night shift (**Figure 1**).

The HORMONIT study was approved by the Parc de Salut Mar Clinical Research Ethics committee (#2015/6351) and after receiving a leaflet with study information, all participants provided written informed consent. A total of 71 men volunteered for the study. After reviewing eligibility criteria (male, rotating shift worker with fixed 3-week shift rotations, without a prior diagnosis of cancer, between the ages of 18-65), 7 were found to be ineligible and 8 withdrew prior to study start, resulting in 56 participants who were enrolled in the study. A total of 51 participants completed the full study protocol, including blood samples extractions in EDTA tubes which were processed and stored at -80°C to be used in the measurement of cytokine concentrations in plasma. Therefore, the following analyses include data from 51 participants.

### Exposures

At the starting morning shift, participants were interviewed and data on demographics, work-related information, smoking, alcohol and caffeine consumption, dietary habits, medical history and medication use, information on time and quality of sleep, and information on chronotype were collected. In addition to questionnaire-collected data, each participant was given a wrist worn actigraphy device the size of a wrist watch (*Actigraph GT3X+, USA*) capable of measuring triaxial acceleration (41). From the actigraphy device, daily activity and sleep were assessed.

### Outcomes

We measured a panel of 30 cytokines, chemokines and growth factors in 196 plasma samples by Luminex using the Cytokine Human Magnetic 30-Plex Panel LHC6003M from Life Technologies™. This panel included the following analytes which are involved in inflammatory or immune responses: epidermal growth factor (EGF), eotaxin, fibroblast growth factor (FGF), granulocyte colony-stimulating factor (G-CSF), granulocyte-macrophage colony-stimulating factor (GM-CSF), hepatocyte growth factor (HGF), interferon-alpha (IFN-α), IFN-γ, interleukin-1 receptor agonist (IL-1RA), IL-1β, IL-2, IL-2R, IL-4, IL-5, IL-6, IL-7, IL-8, IL-10, IL-12(p40/p70), IL-13, IL-15, IL-17, IFN–γ induced protein-10 (IP-10), monocyte chemoattractant protein-1 (MCP-1), monokine induced by IFN–γ (MIG), macrophage inflammatory protein-1α (MIP-1α), MIP-1β, Regulated upon Activation, Normal T Cell Expressed and Presumably Secreted (RANTES), Tumor necrosis factor (TNF), and vascular endothelial growth factor (VEGF).

Briefly, 25 μL of plasma were tested, applying a modification to the manufacturer’s protocol by using half the volume of all reagents including the standards. This modification was previously tested and showed no difference in assay performance compared to the original protocol and has been used in prior studies (42–44). Each plate included 16 serial dilutions (2-fold) of a standard sample with known concentrations of each analyte, two blanks and three positive controls of high, medium and low concentrations in duplicates, prepared from the standard for quality control purposes. Samples were acquired on a Luminex^®^ 100/200 instrument and analyzed with xPONENT^®^ software 3.1. Concentrations of analytes were obtained by interpolating the median fluorescent intensity (MFI) (after blank MFI subtraction) to a 5-parameter logistic regression curve calculated by xPONENT^®^ and reported as pg/mL. Values below the lower limit of detection (mean of blanks + 2 standard deviations) were assigned half the expected concentration at the lower limit of detection, and values above the upper limit of quantification were assigned twice the expected concentration at the upper limit of quantification. Analytes with ≥30% of data out of the quantification range (imputed) were excluded from the analyses. This resulted in the removal of IL-7, IL-8 and IL-13 from analyses, because many of the results were below the low limit of quantification (LLOQ). Finally, because the distributions of analytes were skewed, we applied a log_10_ transformation to the data.

### Covariates

We examined within-person variations in immune marker concentrations and thus adjustment for many covariates traditionally adjusted for in shift work circadian rhythm studies was not required. However, variation in daylight hours is known to impact circadian rhythms (45, 46). Because biological samples were taken on a night shift and a day shift working day, that in some cases differed by several weeks, we adjusted analyses for length of daylight using values available from the National Oceanic and Atmospheric Association calculator and inputting the latitude 41^0^ 230 N and longitude 2^0^ 100 E for Barcelona (47). We also included sleep in secondary analyses. First, we assessed sleep duration on both a day and night shift using actigraphy data. Second, sleep quality was assessed using participant responses to questionnaires. To define chronotype in our study, sleep duration was calculated from patient reported sleep onset and offset using the Munich Chronotype Questionnaire for shift workers (MCTQShift) (48). Participants responded to the MCTQShift at the beginning of a day shift and at the beginning of a night shift. Chronotype (MSF_corr_) was estimated as the mid-sleep time on free days (MSF = sleep onset on free day + [sleep duration on free day/2]), corrected for oversleep on free days compared to working days (MSF_corr_ = MSF – [sleep duration on free day-sleep duration on a working day/2]). We assessed chronotype as a categorical variable where categories were built using tertiles of the distribution in our population (morning type: MSF < 4:00 h, neither type: MSF [04:00– 04:50] h, evening type: MSF > 04:50 h) (49), this was done because the standard cut-offs are designed to describe a general population. In our study, we have a specific population of male rotating night shift workers, so the MSF and subsequent chronotype is expected to be different.

### Statistical analyses

First, to visually compare immune markers sampled at the same time between day and night shifts, we generated boxplots presenting the concentrations of immune markers sampled at 06:00 h (corresponding to the end of the night shift and the beginning of the day shift) during each shift and compared them using paired t-tests. Then, we applied linear mixed models to examine intra-individual associations between shift work and log_10_ concentration of each cytokine, chemokine and growth factor comparing samples taken at 06:00 h. Models were adjusted for hours of daylight. We also examined results from fully-adjusted models that included a) sleep duration and b) sleep quality, because sleep may result in changes in cytokine levels through a different pathway and we wanted to isolate the impact of shift on cytokine levels irrespective of sleep. Furthermore, because some participants were sampled during the 2^nd^ week and others during the 3^rd^ week of a given rotation, which could influence the extent of adaptation to the shift schedule and the extent of change in immune marker levels, we conducted an analysis that included a variable for “shift intensity” into the mixed model, which described how many days into a 3-week rotation participants were at the time of blood sampling. In addition, we evaluated if the shift work effect on immune markers concentrations was modified by participant chronotype. To examine this effect modification, we added an interaction term into our linear mixed models, and then used likelihood ratio tests to determine if the interaction term was significant.

Second, we conducted an exploratory factor analysis, which is a variable reduction method that attempts to identify the smallest number of factors that explain the covariation observed among a set of variables (50). Factor analysis is a useful approach for examining immune markers, which are generally highly correlated (51). This method allowed us to assess the association and correlation between different cytokines (pro- and anti-inflammatory), chemokines and growth factors and how these are influenced by night shift work. Bartlett’s test of sphericity was applied to ensure immune analytes were correlated, and that the correlation matrix was not random (52). Using oblique rotation, we identified common factors for all measured analytes, focusing on factors that explained 10% or more of the total variance (prior to rotation) in the study population. Then, we examined whether these factors were associated with shift work using linear mixed models.

Finally, to assess if immune markers were influenced by the time of sampling, we compared analyte concentrations from the same individual at the start and end of each shift (work point), separately for the day and night shifts. We also examined a linear mixed model that included cytokines for all for time points: before and after on the day shift and before and after on the night shift to see if levels were different due to shift differences (night versus day) or based on time point differences (before versus after shift). All analyses were performed using Stata, StataCorp. 2019. Stata Statistical Software: Release 16. College Station, TX: StataCorp LLC.

## Results

Participants had a mean age of 38 (± SD 9) years. The majority had a body mass index (BMI) ≥25 (53%), 38% were current smokers, and participants on average had worked night shifts for 10 (± SD 6) years. On average, 45 (± SD 43) days elapsed between sampling time points, which resulted in an average of 72 (± SD 67) minutes of daylight difference between the sampling time points (**Table 1**). For day shift samples, 14% of participants were sampled during the 2^nd^ week of rotation, while the majority (86%) of participants were sampled during the 3^rd^ week of rotation. For night shift samples the corresponding numbers were 20% and 80%. Samples were collected for participants at a median of 16 days (IQR 16-18) into the 21-day shift rotation and 16 days (IQR 16-17) into the night shift rotation. Furthermore, participants had worked a median of 2 (IQR 2-3) consecutive days prior to when they day shift samples were collected and a median of 2 (IQR 1-2) days prior to when the night shift samples were collected.

**Table 1:** Characteristics of study population (N=51).

Variable	Mean (SD) or %^a^
Age, years	38 (9)
Height, cm	174 (7)
Weight, Kg	79 (13)
BMI	
<25	47%
25-30	35%
≥30	18%
Smoking	
Never	42%
Former	20%
Current	38%
Education	
Primary	27%
Professional	73%
Chronotype	
Morning	34%
Neither	33%
Evening	33%
Physical activity level of work	
Sedentary	4%
Low intensity	14%
Moderately active	49%
Very active	33%
Actigraphy	
Duration of sleep on day shift, median (IQR)	5.78 (5.03, 6.57)
Duration of sleep on night shift, median (IQR)	5.85 (5.08, 6.65)
Bad quality of sleep	
Between day shifts	38%
Between night shifts	45%
Cumulative duration of shift work history, years^b^	10 (6)
Diagnosed with a chronic disease^c^	25%
Currently use medication^d^	22%
Number of days into shift at the time of collection, median (IQR)	
Day	16 (16-18)
Night	16 (16-17)
Number of consecutive days worked prior to collection, median (IQR)	
Day	2 (2-3)
Night	2 (1-2)
Average number of days between sampling time points	45 (43)
Average minutes of daylight difference between day and night shift	72 (67)

a Mean (SD) or % reported unless otherwise noted.

b Cumulative duration of shift work history was missing for 15 (30%) participants.

c Reported chronic conditions include: anemia, asthma or allergy, cholesterol, hepatitis C, hypertension, attention deficit disorder, uric acid, anxiety.

d Medications reported include: use of aspirin, hypnotics, melatonin or a sedative.

Results are presented in terms of different functional groups for inflammatory responses, broken down into: pro-inflammatory, anti-inflammatory, Th1-type cytokines (which promote macrophage activation, nitric oxide production, and cytotoxic T lymphocyte proliferation, leading to the phagocytosis and destruction of microbial pathogens), Th2-type cytokines (which activate and maintain the antibody-mediated immune response against extracellular parasites, bacteria, allergens, and toxins, activate eosinophils, and inhibit several macrophage functions, thus providing phagocyte-independent protective responses), Th17-type cytokines (which are relevant in the defense against microbial infections, particularly against extracellular bacteria and mucosal fungi), chemokines and growth factors. From descriptive analyses examining cytokine, chemokine and growth factor concentrations in the same individual and at the same time (06:00 h) at the start of a day shift and at the end of a night shift, we found lower concentrations of several analytes at the end of the night shift (**Supplemental Figure 1**).

Next, linear mixed models showed 15 analytes with intra-individual differences in their concentrations depending on shift when comparing samples collected at 06:00 h at the end of a night shift and the same hour at the start of a day shift. All 15 analytes showed lower concentrations at the end of the night shift working day: the pro-inflammatory cytokines TNF-α and IL-2R; the anti-inflammatory cytokine IL-1RA; the Th1-type cytokine IL-12; the Th2-type cytokine IL-4; and the Th17-type cytokine IL-17. Lower concentrations of several chemokines were also observed at the night shift compared to the day shift, including lower levels of IP-10, MIP-1α, MIP-1β, and RANTES. Finally, several growth factors were also lower at the end of night shift compared to the day shift including EGF, G-CSF, HGF, VEGF and FGF (**Table 2**). When examining results from the two additional sets of models that included sleep variables (duration and quality), results were very similar to the results from our primary models (**Supplemental Table 1**). When examining results from the model including information on shift intensity at the time of sampling, estimates were similar to the primary models, but more pronounced, suggesting that a higher intensity of shift work may be associated with larger differences in immune marker concentrations (**Supplemental Table 2**).

**Table 2:** Association between shift work and cytokines concentrations (log_10_ pg/mL) from mixed models comparing morning sample at the end of a night shift vs. morning sample at the start of a day shift (06:00 h).

Analyte	β^a^	95% confidence interval		Raw analyte levels (log_10_ pg/mL), (mean (SD))	
				Day	Night
*Pro-inflammatory*					
IL-1β	-0.06	-0.17	0.04	0.89 (0.83)	0.86 (0.68)
TNF-α	-0.09	-0.15	-0.03	0.52 (0.41)	0.42 (0.40)
IL-2R	-0.06	-0.11	-0.02	2.09 (0.35)	2.03 (0.34)
IFN-α	-0.03	-0.08	0.02	1.27 (0.34)	1.22 (0.30)
*Anti-inflammatory*					
IL-10	0.01	-0.07	0.09	0.91 (0.74)	0.92 (0.73)
IL-1RA	-0.24	-0.34	-0.14	2.81 (0.37)	2.53 (0.51)
*Th1*					
IFN-γ	0.02	-0.05	0.09	-0.15 (0.43)	-0.11 (0.32)
IL-12	-0.03	-0.06	0.00	1.68 (0.28)	1.67 (0.25)
IL-2	-0.05	-0.10	0.00	0.35 (0.43)	0.31 (0.39)
IL-15	-0.06	-0.12	0.01	1.42 (0.65)	1.38 (0.59)
*Th2*					
IL-4	-0.11	-0.19	-0.03	0.82 (0.52)	0.72 (0.48)
IL-5	-0.03	-0.09	0.02	-0.09 (0.54)	-0.11 (0.54)
IL-6	-0.05	-0.11	0.01	0.63 (0.55)	0.62 (0.49)
*Th17*					
IL-17	-0.13	-0.25	0.00	-0.14 (0.74)	-0.21 (0.76)
*Chemokines *					
IP-10	-0.05	-0.09	-0.01	1.77 (0.22)	1.67 (0.23)
MCP-1	0.01	-0.02	0.04	2.57 (0.20)	2.57 (0.17)
MIP-1α	-0.04	-0.08	0.00	1.42 (0.32)	1.36 (0.32)
MIP-1β	-0.06	-0.11	-0.02	1.51 (0.37)	1.41 (0.35)
EOTAXIN	0.02	-0.03	0.07	2.07 (0.31)	2.10 (0.22)
RANTES	-0.33	-0.43	-0.23	3.26 (0.52)	2.95 (0.40)
MIG	-0.05	-0.14	0.05	1.37 (0.48)	1.35 (0.56)
*Growth factors*					
EGF	-0.17	-0.25	-0.08	0.74 (0.59)	0.57 (0.50)
G-CSF	-0.08	-0.15	-0.01	1.56 (0.32)	1.43 (0.39)
GM-CSF	-0.04	-0.10	0.03	0.21 (0.71)	0.20 (0.70)
HGF	-0.06	-0.11	-0.01	1.82 (0.34)	1.77 (0.33)
VEGF	-0.10	-0.18	-0.02	0.44 (0.52)	0.30 (0.64)
FGF	-0.08	-0.15	-0.01	1.35 (0.50)	1.25 (0.54)

a Models adjusted for hours of daylight.

The factor analysis elucidated three factors which combined explained 81% of the total variance of the analyte concentrations before rotation. Factor 1 explained 57% of the variance, Factor 2 explained 14% of the variance and Factor 3 explained 10% of the variance. The loading values after applying oblique rotation are provided in **Table 3**. The factor loading values express the correlation between a given analyte and the factor. Analytes with factors loadings of 0.60 or higher were considered to be major components of the factor. Factor 1 comprised cytokines IL-1β, IL-2, IL-15; chemokines MIP-1α, MIP-1β; and growth factors EGF, FGF. Factor 2 comprised anti-inflammatory cytokine IL-10 and the growth factor GM-CSF. Factor 3 comprised the Th1 cytokine IL-12. When examining associations between the factors and the shift work and sampling time, Factor 1 was associated with shift, with lower levels of the factor during the 06:00 h night shift sample (coefficient - 0.17, 95%CI -0.32, -0.01) compared to the referent 06:00 h day shift sample. Factor 3 was also associated with the shift work, with lower levels of the factor during the 06:00 h night shift sample (coefficient - 0.22, 95%CI -0.38, -0.06) compared to the referent 06:00 h day shift sample (**Table 4**). Factor 2 was not associated with night shift. None of the factors were associated with the other times of sampling (end of day shift or start of night shift). These results indicate that night shift work reduces several cytokine concentrations compared to day shift work when samples are taken at the same time of the day (06:00 h).

**Table 3:** Rotated factor loading values of the 27 analytes in the 3 main factors elucidated in factor analysis after applying oblique rotation.

Analyte	Factor 1	Factor 2	Factor 3
*Proinflammatory*			
IL-1β	0.72	0.00	0.03
TNF-α	0.39	0.05	-0.13
IL-2R	0.00	0.01	0.06
IFN-α	0.51	0.01	-0.07
*Anti-inflammatory*			
IL-10	0.04	0.97	-0.10
IL1-RA	0.06	0.12	0.05
*Th1*			
IFN-γ	-0.01	0.03	0.09
IL-12	0.20	0.03	0.73
IL-2	0.98	0.00	-0.05
IL-15	0.96	0.05	-0.05
*Th2*			
IL-4	0.47	-0.02	0.03
IL-5	-0.13	0.15	0.08
IL-6	0.41	0.21	-0.09
*Th17*			
IL-17	0.10	0.02	-0.15
*Chemokines*			
IP-10	0.00	-0.07	0.00
MCP-1	0.18	0.01	0.15
MIP-1α	0.86	-0.04	0.02
MIP-1β	0.66	-0.03	-0.03
EOTAXIN	-0.03	-0.02	-0.10
RANTES	-0.03	-0.09	-0.04
MIG	-0.04	-0.02	-0.05
*Growth factors*			
EGF	0.83	0.02	0.07
G-CSF	-0.07	-0.03	0.02
GM-CSF	0.01	0.88	0.11
HGF	0.58	0.00	0.20
VEGF	0.09	-0.02	0.17
FGF	0.92	-0.04	0.13
Eigenvalues	11.05	2.64	2.00
Proportion of variance before rotation	0.57	0.14	0.10

Unique variances, eigenvalues and proportion of variance are shown.

**Table 4:** Associations of the 3 factors, obtained through factor analysis, with shift work and time of sampling.

	β	95%CI
Factor 1		
06:00 (day shift start)	Ref.	Ref.
14:00 (day shift end)	0.10	(-0.05, 0.26)
22:00 (night shift start)	-0.08	(-0.23, 0.07)
06:00 (night shift end)	-0.17	(-0.32, -0.01)
Factor 2		
06:00 (day shift start)	Ref.	Ref.
14:00 (day shift end)	0.02	(-0.11, 0.14)
22:00 (night shift start)	0.04	(-0.09, 0.16)
06:00 (night shift end)	0.00	(-0.12, 0.12)
Factor 3		
06:00 (day shift start)	Ref.	Ref.
14:00 (day shift end)	0.11	(-0.06, 0.27)
22:00 (night shift start)	-0.07	(-0.24, 0.09)
06:00 (night shift end)	-0.22	(-0.38, -0.06)

We did not find any effect modification by chronotype on the cytokine concentrations (**Supplemental Table 3**). When we compared analyte concentrations at the start versus at the end of a given shift, we observed several analytes at a slightly higher level at the end of the day shift: IL-2R, IL-1RA, IL-12, IL-4, RANTES and EGF, and only two with a lower level: IP-10 and MCP-1 (**Supplemental Table 4**). On the contrary, several analytes slightly decreased after the night shift: TNF-α, IL-1RA, IP-10, MIP-1α, RANTES, EGF and VEGF, and only two increased IL-6 and MCP-1 (**Supplemental Table 4**). However, the magnitude of overall differences between working points within shifts were minimal compared to differences between shifts. Finally, when we examined results based on shift differences and time of sampling (after versus before shift), we observed that differences were driven by shift differences (night versus day) rather than by time point differences (after versus before the shift) (**Supplemental Table 5**).

## Discussion

Our results indicate that 2 or 3 weeks of night shift work alters the plasma concentration of several immune markers. We found that night shift work was associated with lower concentrations of several immune markers including cytokines TNF-α, IL-2R, IL1-RA, IL-12, IL-4 and IL-17 as well as chemokines IP-10, MIP-1α, MIP-1β and RANTES, and growth factors G-CSF, FGF, EGF, HGF and VEGF. Several of these analytes, plus others, were also identified by a factor analysis as contributing to two factors inversely associated to the night shift work, suggesting again lower concentrations of several immune markers in the night shift. This overall decrease in immune marker concentrations in the night shift compared to the day shift could be due to a disruption of the circadian rhythm. The majority of the cytokines and chemokines that were lower among night shift workers in our study are proinflammatory in nature, indicating a dampened immune response that could be related to the increased risk of infections and cancer reported among night shift workers (20, 25). This suppressed immune status could also dampen immune responses to vaccines. Indeed, a recent study showed that night shift workers have weaker humoral response to a meningococcal conjugate vaccine compared to day shift workers (53). Also, relevant to current events, it has even been postulated that night shift work may increase susceptibility to the 2019 novel coronavirus (COVID-19) (54).

Among the analytes identified in the factor analysis with lower concentrations in the night shift were the cytokines IL-1β and IL-15, the Th1 cytokines IL-2 and IL-12, the chemokines MIP-1α and MIP-1β and the growth factors EGF and FGF. The cytokines IL-1β and IL-15 are important in the development of inflammatory and protective immune responses to microbes and cancer (55, 56). The Th1 cytokines IL-2 and IL-12 are also important in the defense against infections and cancer (57), involved in the development of memory responses against pathogens, and in the control of autoimmunity (58). The chemokines MIP-1α and MIP-1β have chemotactic and pro-inflammatory effects in addition to contributing to the defense of infections (59, 60). Finally, the growth factors EGF and FGF are important for the repair of injured tissues during inflammatory processes (61).

Results from prior studies also suggest that night shift work alters endogenous cytokines. Liu et al. found that relative to participants in a simulated day shift condition, those in the simulated night shift condition (sample size=14) had lower levels of TNF-α (62). Loef et al. also examined cytokine levels among night shift workers (n=254) compared to non-night shift workers (n=57) (63). Authors reported a higher level of MIP-1β among night shift workers (β 1.39, 95%-CI=1.10–1.77), but found no change in the levels of several other cytokines. In our study, we found that MIP-1β was at lower concentrations during the night shift, and additional analytes showed also altered. These differences between studies are likely the result of differences in the study population, the methods for cytokine of assessment, the comparison of between versus within-person comparisons, the degree of control of confounding factors, and the timing of biologic sample collection.

Using available data, we were able to examine how sleep duration and quality among our population may impact cytokine levels. Within our population, we did not see large differences between the sleep duration or quality between the two shifts. This may be because in such slowly rotating shift schedules, with several consecutive nights, workers are more likely to adapt to the shift work schedule and thus sleep longer and better. This lack of variability may be driving the fact that we did not see any noteworthy changes in model results from models that included adjustment for sleep compared to the primary analyses. This suggests that within our population, the resulting changes in cytokine levels between the night and day shift are not driven by a sleep-related pathway but rather by circadian rhythm disruption. Future studies that collect more detailed information on sleep quality may be needed to examine the sleep-specific cytokine pathway in more depth.

This study had several strengths including minimizing the effects of confounding by comparing within participant immune analyte levels during the night versus the day shift. We were also able to adjust for daylight hours, which influenced our results, indicating that this is an important variable to consider in related studies. Furthermore, we analyzed a panel of 27 analytes, while prior studies have examined only a few analytes at a time, allowing for a broader view of the impact of night shift work on cellular markers. In addition, we measured cytokine concentrations at multiple time points during each shift. This provides more information than relying on cytokine concentrations assessed at a single time point, and accounts for diurnal variation of biomarkers. Finally, we were able to examine how sleep influenced the associations between night shift and cytokine levels, allowing us to examine if the resulting changes in cytokine levels were due to night shift or acute sleep deprivation in our sample. However, this study also has limitations including the small sample size, which was mitigated to some extent by having repeated measurements for each participant. In addition, although we took blood at 2 time points during each shift, having more information on immune marker levels during the rest of the 24 h day would have been useful to better assess the effect of night shift on the circadian rhythm of these analytes. It would have been interesting to analyze changes in the cellular populations between day and night shifts to complement cytokines data, however cellular samples were not available from the study participants. Another potential limitation of the study design we applied that minimizes confounding, could be that the selected immunoprotein panel also reflects long term effects of work rotation on the immunological state rather than only reflecting changes related to the slow (3-week) rotation. As shown in a previous paper analyzing patterns of melatonin (aMT6s, a major melatonin metabolite) in the same population, workers were well adapted to their shift at the time we sampled (64).

In summary, we found overall lower cytokine, chemokine and growth factor levels during night shift work, suggesting a suppressed immune status among night shift workers that could be associated with a higher risk of infection and cancer reported among this population. Our findings suggest more research and subsequent policy recommendations should be made that would encourage optimum health practices for minimizing the negative health impacts of night shift work on the immune response. Future work that examines in larger samples how cytokine levels among night shift workers are influenced by altered hormone responses would increase our understanding of the mechanisms underlying the increased susceptibility of these workers to infections and chronic diseases.

## Data availability statement

The raw data supporting the conclusions of this article will be made available by the authors, without undue reservation.

## Ethics statement

The studies involving human participants were reviewed and approved by the Parc de Salut Mar Clinical Research Ethics committee (#2015/6351) and after receiving a leaflet with study information, all participants provided written informed consent.

## Author contributions

BH, RA, KP, CD, GM, MK conceived and designed the analysis. RA, AE, GC-V, JN, PF, AT, CD, GM and MK collected the data. BH contributed to the data analysis. BH and RA wrote the paper. All authors reviewed the results and approved the final version of the manuscript.

## Funding

The studies involving human participants were reviewed and approved by the Parc de Salut Mar Clinical Research Ethics committee (#2015/6351) and after receiving a leaflet with study information, all participants provided written informed consent.

## Acknowledgments

The authors thank Emilia Molinero, Mayte Martín Bustamante and Elena Juanola Pages from the Generalitat de Catalunya for all of their support in planning this study. The authors also thank the study participants for their important contributions.

## Conflict of interest

JN, PF and AT work at the Occupational Health service of the car factory, which was the setting of the present study. At the HORMONIT study working group they express their own views and do not represent the company.

The remaining authors declare that the research was conducted in the absence of any commercial or financial relationships that could be construed as a potential conflict of interest.

## Publisher’s note

All claims expressed in this article are solely those of the authors and do not necessarily represent those of their affiliated organizations, or those of the publisher, the editors and the reviewers. Any product that may be evaluated in this article, or claim that may be made by its manufacturer, is not guaranteed or endorsed by the publisher.
