# Supplementary material for: Disruption of cellular immune response among male rotating night shift workers in Spain– The HORMONIT study
Source: Front Immunol. 2022 Sep 2;13:776917. doi: 10.3389/fimmu.2022.776917 (PMC9478612; doi:10.3389/fimmu.2022.776917)
Supplement: Supplementary file 1 [file Table_1.docx]

**Supplemental Materials:**

Supplemental Table 1: Association between shift work and cytokines concentrations (log_10_ pg/mL) from mixed models including adjustment for sleep comparing morning sample at the end of a night shift vs. morning sample at the start of a day shift (06:00 h)

|  | **Including duration of sleep** | | | **Including quality of sleep** | | |
| --- | --- | --- | --- | --- | --- | --- |
| **Analyte** | **β^a^** | **Confidence interval** | | **β^a^** | **Confidence interval** | |
| *Pro-inflammatory* | | | | | | |
| IL-1β | -0.10 | -0.19 | -0.01 | -0.10 | -0.19 | -0.01 |
| TNF-α | -0.09 | -0.15 | -0.03 | -0.09 | -0.15 | -0.03 |
| IL-2R | -0.08 | -0.12 | -0.04 | -0.08 | -0.12 | -0.04 |
| IFN-α | -0.03 | -0.07 | 0.02 | -0.03 | -0.07 | 0.02 |
| *Anti-inflammatory* | | | | | | |
| IL-10 | -0.01 | -0.09 | 0.07 | -0.02 | -0.10 | 0.07 |
| IL1-RA | -0.22 | -0.31 | -0.13 | -0.22 | -0.31 | -0.13 |
| *Th1* | | | | | | |
| IFN-γ | 0.00 | -0.08 | 0.07 | 0.00 | -0.08 | 0.08 |
| IL-12 | -0.03 | -0.06 | 0.00 | -0.03 | -0.06 | 0.00 |
| IL-2 | -0.05 | -0.09 | 0.00 | -0.05 | -0.09 | 0.00 |
| IL-15 | -0.06 | -0.11 | -0.01 | -0.06 | -0.11 | -0.01 |
| *Th2* | | | | | | |
| IL-4 | -0.09 | -0.17 | -0.02 | -0.10 | -0.17 | -0.02 |
| IL-5 | -0.04 | -0.10 | 0.03 | -0.04 | -0.10 | 0.02 |
| IL-6 | -0.07 | -0.12 | -0.01 | -0.07 | -0.13 | -0.01 |
| *Th17* | | | | | | |
| IL-17 | -0.15 | -0.27 | -0.02 | -0.15 | -0.28 | -0.02 |
| *Chemokines* | | | | | | |
| IP-10 | -0.05 | -0.10 | -0.01 | -0.05 | -0.10 | -0.01 |
| MCP-1 | 0.01 | -0.02 | 0.04 | 0.01 | -0.02 | 0.03 |
| MIP-1α | -0.04 | -0.07 | 0.00 | -0.04 | -0.07 | 0.00 |
| MIP-1β | -0.08 | -0.12 | -0.04 | -0.08 | -0.12 | -0.04 |
| EOTAXIN | 0.01 | -0.02 | 0.05 | 0.01 | -0.02 | 0.05 |
| RANTES | -0.32 | -0.43 | -0.21 | -0.32 | -0.43 | -0.21 |
| MIG | -0.04 | -0.15 | 0.06 | -0.04 | -0.15 | 0.06 |
| *Growth factors* | | | | | | |
| EGF | -0.14 | -0.22 | -0.06 | -0.14 | -0.22 | -0.06 |
| G-CSF | -0.09 | -0.16 | -0.02 | -0.09 | -0.16 | -0.02 |
| GM-CSF | -0.06 | -0.13 | 0.01 | -0.06 | -0.13 | 0.01 |
| HGF | -0.06 | -0.10 | -0.02 | -0.06 | -0.10 | -0.02 |
| VEGF | -0.09 | -0.16 | -0.03 | -0.09 | -0.16 | -0.03 |
| FGF | -0.09 | -0.15 | -0.02 | -0.09 | -0.15 | -0.03 |

Supplemental Table 2: Association between shift work and cytokines concentrations (log_10_ pg/mL) from mixed models including adjustment for night shift intensity comparing morning sample at the end of a night shift vs. morning sample at the start of a day shift (06:00 h)

|  | **Results from primary analysis** | | | **Results from sensitivity analysis with inclusion of night shift intensity** | | |
| --- | --- | --- | --- | --- | --- | --- |
| **Analyte** | **β^a^** | **95% confidence interval** | | **β^a^** | **95% confidence interval** | |
|  |  |  | |  |  |  |
| *Pro-inflammatory* | |  |  |  |  |  |
| IL-1β | -0.06 | -0.17 | 0.04 | -0.12 | -0.21 | -0.02 |
| TNF-α | -0.09 | -0.15 | -0.03 | -0.12 | -0.18 | -0.06 |
| IL-2R | -0.06 | -0.11 | -0.02 | -0.08 | -0.12 | -0.04 |
| IFN-α | -0.03 | -0.08 | 0.02 | -0.07 | -0.11 | -0.02 |
| *Anti-inflammatory* | |  |  |  |  |  |
| IL-10 | 0.01 | -0.07 | 0.09 | -0.02 | -0.11 | 0.07 |
| IL-1RA | -0.24 | -0.34 | -0.14 | -0.25 | -0.36 | -0.14 |
| *Th1* |  |  |  |  |  |  |
| IFN-γ | 0.02 | -0.05 | 0.09 | -0.01 | -0.09 | 0.07 |
| IL-12 | -0.03 | -0.06 | 0.00 | -0.04 | -0.07 | -0.01 |
| IL-2 | -0.05 | -0.10 | 0.00 | -0.07 | -0.12 | -0.02 |
| IL-15 | -0.06 | -0.12 | 0.01 | -0.11 | -0.17 | -0.05 |
| *Th2* |  |  |  |  |  |  |
| IL-4 | -0.11 | -0.19 | -0.03 | -0.15 | -0.24 | -0.06 |
| IL-5 | -0.03 | -0.09 | 0.02 | -0.07 | -0.14 | -0.01 |
| IL-6 | -0.05 | -0.11 | 0.01 | -0.07 | -0.13 | -0.02 |
| *Th17* |  |  |  |  |  |  |
| IL-17 | -0.13 | -0.25 | 0.00 | -0.18 | -0.29 | -0.06 |
| *Chemokines* |  |  |  |  |  |  |
| IP-10 | -0.05 | -0.09 | -0.01 | -0.06 | -0.11 | -0.01 |
| MCP-1 | 0.01 | -0.02 | 0.04 | -0.02 | -0.05 | 0.01 |
| MIP-1α | -0.04 | -0.08 | 0.00 | -0.07 | -0.11 | -0.03 |
| MIP-1β | -0.06 | -0.11 | -0.02 | -0.09 | -0.13 | -0.04 |
| EOTAXIN | 0.02 | -0.03 | 0.07 | 0.00 | -0.03 | 0.04 |
| RANTES | -0.33 | -0.43 | -0.23 | -0.33 | -0.45 | -0.21 |
| MIG | -0.05 | -0.14 | 0.05 | -0.11 | -0.23 | 0.00 |
| *Growth factors* |  |  |  |  |  |  |
| EGF | -0.17 | -0.25 | -0.08 | -0.19 | -0.28 | -0.10 |
| G-CSF | -0.08 | -0.15 | -0.01 | -0.11 | -0.18 | -0.03 |
| GM-CSF | -0.04 | -0.10 | 0.03 | -0.08 | -0.16 | -0.01 |
| HGF | -0.06 | -0.11 | -0.01 | -0.08 | -0.13 | -0.04 |
| VEGF | -0.10 | -0.18 | -0.02 | -0.14 | -0.21 | -0.06 |
| FGF | -0.08 | -0.15 | -0.01 | -0.13 | -0.20 | -0.06 |

Supplemental Table 3. Results from likelihood- ratio test to examine if there is an effect modification by chronotype for analytes’ concentrations

| **P-value from likelihood-ratio test** | |
| --- | --- |
| **Analyte** | **Chronotype** |
| *Proinflammatory* |  |
| IL-1β | 0.83 |
| TNF-α | 0.65 |
| IL-2R | 0.51 |
| IFN-α | 0.96 |
| *Anti-inflammatory* |  |
| IL-10 | 0.96 |
| IL1-RA | 0.80 |
| *Th1* |  |
| IFN-γ | 0.54 |
| IL-12 | 0.83 |
| IL-2 | 0.54 |
| IL-15 | 0.85 |
| *Th2* |  |
| IL-4 | 0.71 |
| IL-5 | 0.93 |
| IL-6 | 0.92 |
| *Th17* |  |
| IL-17 | 0.60 |
| *Chemokines* |  |
| IP-10 | 0.91 |
| MCP-1 | 0.99 |
| MIP-1α | 0.79 |
| MIP-1β | 0.90 |
| EOTAXIN | 0.70 |
| RANTES | 0.20 |
| MIG | 0.94 |
| *Growth factors* |  |
| EGF | 0.46 |
| G-CSF | 0.94 |
| GM-CSF | 0.86 |
| HGF | 0.79 |
| VEGF | 0.98 |
| FGF | 0.54 |

Supplemental Table 4. Comparison of analyte concentrations (log_10_ (pg/mL)) by time of sampling in the day and night shifts

|  | **Day shift** | | | |  | **Night shift** | | | |  |
| --- | --- | --- | --- | --- | --- | --- | --- | --- | --- | --- |
|  | **Start of shift (06:00 h)** | | **End of shift (14:00 h)** | |  | **Start of shift (22:00 h)** | | **End of shift (06:00 h)** | |  |
| **Analyte** | **mean** | **SD** | **mean** | **SD** | **t-test** | **mean** | **SD** | **mean** | **SD** | **t-test** |
| *Pro-inflammatory* | | | | | | | | | | |
| IL-1β | 0.89 | 0.83 | 1.03 | 0.45 | 0.12 | 0.95 | 0.50 | 0.86 | 0.68 | 0.13 |
| TNF-α | 0.52 | 0.41 | 0.59 | 0.33 | 0.06 | 0.51 | 0.33 | 0.42 | 0.40 | 0.03 |
| IL-2R | 2.09 | 0.35 | 2.17 | 0.25 | 0.01 | 2.07 | 0.31 | 2.03 | 0.34 | 0.13 |
| IFN-α | 1.27 | 0.34 | 1.31 | 0.28 | 0.19 | 1.25 | 0.25 | 1.22 | 0.30 | 0.08 |
| *Anti-inflammatory* | | | | | | | | | | |
| IL-10 | 0.91 | 0.74 | 0.93 | 0.71 | 0.59 | 0.98 | 0.67 | 0.92 | 0.73 | 0.06 |
| IL1-RA | 2.81 | 0.37 | 2.91 | 0.35 | 0.05 | 2.70 | 0.41 | 2.53 | 0.51 | 0.01 |
| *Th1* | | | | | | | | | | |
| IFN-γ | -0.15 | 0.43 | -0.11 | 0.30 | 0.36 | -0.11 | 0.36 | -0.11 | 0.32 | 0.93 |
| IL-12 | 1.68 | 0.28 | 1.72 | 0.23 | 0.03 | 1.68 | 0.23 | 1.67 | 0.25 | 0.91 |
| IL-2 | 0.35 | 0.43 | 0.40 | 0.37 | 0.13 | 0.34 | 0.37 | 0.31 | 0.39 | 0.46 |
| IL-15 | 1.42 | 0.65 | 1.49 | 0.55 | 0.18 | 1.38 | 0.59 | 1.38 | 0.59 | 0.58 |
| *Th2* |  |  |  |  |  |  |  |  |  |  |
| IL-4 | 0.82 | 0.52 | 0.91 | 0.40 | 0.03 | 0.79 | 0.45 | 0.72 | 0.48 | 0.07 |
| IL-5 | -0.09 | 0.54 | -0.08 | 0.55 | 0.80 | -0.10 | 0.53 | -0.11 | 0.54 | 0.69 |
| IL-6 | 0.63 | 0.55 | 0.65 | 0.51 | 0.44 | 0.53 | 0.51 | 0.62 | 0.49 | 0.00 |
| *Th17* |  |  |  |  |  |  |  |  |  |  |
| IL-17 | -0.14 | 0.74 | -0.05 | 0.66 | 0.34 | -0.20 | 0.83 | -0.21 | 0.76 | 1.00 |
| *Chemokines* | | | | | | | | | | |
| IP-10 | 1.77 | 0.22 | 1.71 | 0.22 | 0.00 | 1.69 | 0.22 | 1.67 | 0.23 | 0.03 |
| MCP-1 | 2.57 | 0.20 | 2.51 | 0.16 | 0.01 | 2.53 | 0.16 | 2.57 | 0.17 | 0.01 |
| MIP-1α | 1.42 | 0.32 | 1.45 | 0.30 | 0.30 | 1.41 | 0.27 | 1.36 | 0.32 | 0.00 |
| MIP-1β | 1.51 | 0.37 | 1.48 | 0.31 | 0.49 | 1.43 | 0.34 | 1.41 | 0.35 | 0.37 |
| EOTAXIN | 2.07 | 0.31 | 2.07 | 0.22 | 0.97 | 2.09 | 0.22 | 2.10 | 0.22 | 0.66 |
| RANTES | 3.26 | 0.52 | 3.46 | 0.54 | 0.00 | 3.10 | 0.43 | 2.95 | 0.40 | 0.01 |
| MIG | 1.37 | 0.48 | 1.43 | 0.38 | 0.12 | 1.36 | 0.60 | 1.35 | 0.56 | 0.95 |
| *Growth factors* | | | | | | | | | | |
| EGF | 0.74 | 0.59 | 0.86 | 0.39 | 0.04 | 0.69 | 0.46 | 0.57 | 0.50 | 0.01 |
| G-CSF | 1.56 | 0.32 | 1.57 | 0.31 | 0.67 | 1.52 | 0.35 | 1.43 | 0.39 | 0.10 |
| GM-CSF | 0.21 | 0.71 | 0.25 | 0.69 | 0.48 | 0.23 | 0.69 | 0.20 | 0.70 | 0.31 |
| HGF | 1.82 | 0.34 | 1.88 | 0.29 | 0.06 | 1.79 | 0.32 | 1.77 | 0.33 | 0.61 |
| VEGF | 0.44 | 0.52 | 0.52 | 0.36 | 0.09 | 0.41 | 0.52 | 0.30 | 0.64 | 0.02 |
| FGF | 1.35 | 0.50 | 1.39 | 0.46 | 0.36 | 1.31 | 0.50 | 1.25 | 0.54 | 0.12 |

Supplemental Table 5: Association between shift and time of work and cytokines

|  | **Estimates based on shift differences (night versus day)** | | | | **Estimates based on time point differences (levels after work versus before)** | | | |
| --- | --- | --- | --- | --- | --- | --- | --- | --- |
| **Analyte** | **β^a^** | **95% confidence interval** | | **P_shift** | **β^a^** | **95% confidence interval** | | **P_workpoint** |
| *Pro-inflammatory* | | | | | | | | |
| IL-1β | -0.06 | -0.17 | 0.04 | 0.25 | 0.02 | -0.08 | 0.12 | 0.65 |
| TNF-α | -0.09 | -0.15 | -0.03 | **0.00** | -0.01 | -0.06 | 0.04 | 0.65 |
| IL-2R | -0.07 | -0.11 | -0.02 | **0.01** | 0.02 | -0.02 | 0.07 | 0.26 |
| IFN-α | -0.03 | -0.08 | 0.02 | 0.25 | 0.00 | -0.04 | 0.05 | 0.90 |
| *Anti-inflammatory* | | | | | | | | |
| IL-10 | 0.01 | -0.07 | 0.09 | 0.76 | -0.01 | -0.09 | 0.06 | 0.72 |
| IL1-RA | -0.24 | -0.34 | -0.14 | **0.00** | -0.03 | -0.12 | 0.06 | 0.50 |
| *Th1* | | | | | | | | |
| IFN-γ | 0.02 | -0.05 | 0.09 | 0.58 | 0.02 | -0.05 | 0.09 | 0.60 |
| IL-12 | -0.03 | -0.06 | 0.00 | **0.03** | 0.02 | -0.01 | 0.04 | 0.12 |
| IL-2 | -0.05 | -0.10 | 0.00 | 0.06 | 0.01 | -0.04 | 0.05 | 0.77 |
| IL-15 | -0.06 | -0.12 | 0.01 | 0.07 | 0.02 | -0.04 | 0.08 | 0.48 |
| *Th2* | | | | | | | | |
| IL-4 | -0.11 | -0.19 | -0.03 | **0.01** | 0.00 | -0.07 | 0.08 | 0.94 |
| IL-5 | -0.03 | -0.09 | 0.02 | 0.24 | 0.00 | -0.05 | 0.05 | 0.97 |
| IL-6 | -0.05 | -0.11 | 0.01 | 0.08 | 0.06 | 0.00 | 0.11 | **0.03** |
| *Th17* | | | | | | | | |
| IL-17 | -0.13 | -0.25 | 0.00 | **0.04** | 0.04 | -0.08 | 0.15 | 0.52 |
| *Chemokines* | | | | | | | | |
| IP-10 | -0.05 | -0.09 | -0.01 | **0.01** | -0.04 | -0.07 | 0.00 | **0.03** |
| MCP-1 | 0.01 | -0.02 | 0.04 | 0.63 | -0.01 | -0.04 | 0.02 | 0.48 |
| MIP-1α | -0.04 | -0.08 | 0.00 | **0.05** | -0.02 | -0.05 | 0.02 | 0.41 |
| MIP-1β | -0.06 | -0.11 | -0.02 | **0.01** | -0.03 | -0.07 | 0.02 | 0.23 |
| EOTAXIN | 0.02 | -0.03 | 0.07 | 0.39 | 0.00 | -0.04 | 0.05 | 0.98 |
| RANTES | -0.33 | -0.43 | -0.23 | **0.00** | 0.04 | -0.06 | 0.13 | 0.47 |
| MIG | -0.05 | -0.14 | 0.05 | 0.35 | 0.04 | -0.06 | 0.13 | 0.45 |
| *Growth factors* | | | | | | | | |
| EGF | -0.17 | -0.25 | -0.08 | **0.00** | 0.00 | -0.09 | 0.08 | 0.91 |
| G-CSF | -0.08 | -0.15 | -0.01 | **0.03** | -0.04 | -0.10 | 0.03 | 0.28 |
| GM-CSF | -0.04 | -0.10 | 0.03 | 0.29 | 0.00 | -0.06 | 0.06 | 0.99 |
| HGF | -0.06 | -0.11 | -0.01 | **0.01** | 0.02 | -0.02 | 0.07 | 0.27 |
| VEGF | -0.10 | -0.18 | -0.02 | **0.02** | -0.02 | -0.09 | 0.06 | 0.64 |
| FGF | -0.08 | -0.15 | -0.01 | **0.02** | -0.02 | -0.08 | 0.04 | 0.56 |

^a^Models adjusted for hours of daylight
