# Supplementary material for: Disruption of cellular immune response among male rotating night shift workers in Spain– The HORMONIT study
Source: Front Immunol. 2022 Sep 2;13:776917. doi: 10.3389/fimmu.2022.776917 (PMC9478612; doi:10.3389/fimmu.2022.776917)
Supplement: Supplementary file 2 [file DataSheet_1.pdf]

Supplemental Figure 1: Cytokine, chemokine and growth factor concentrations (log_{10} pg/mL) measured in the same individual and at the same time (06:00 h) at the start of a day shift and at the end of a night shift. Boxes depict median log_{10} pg/mL and interquartile ranges (IQR), A paired Wilcoxon rank test was used to assess statistically significant differences in analytes concentrations between shift work. Trajectory points link analytes' concentrations from same individual.

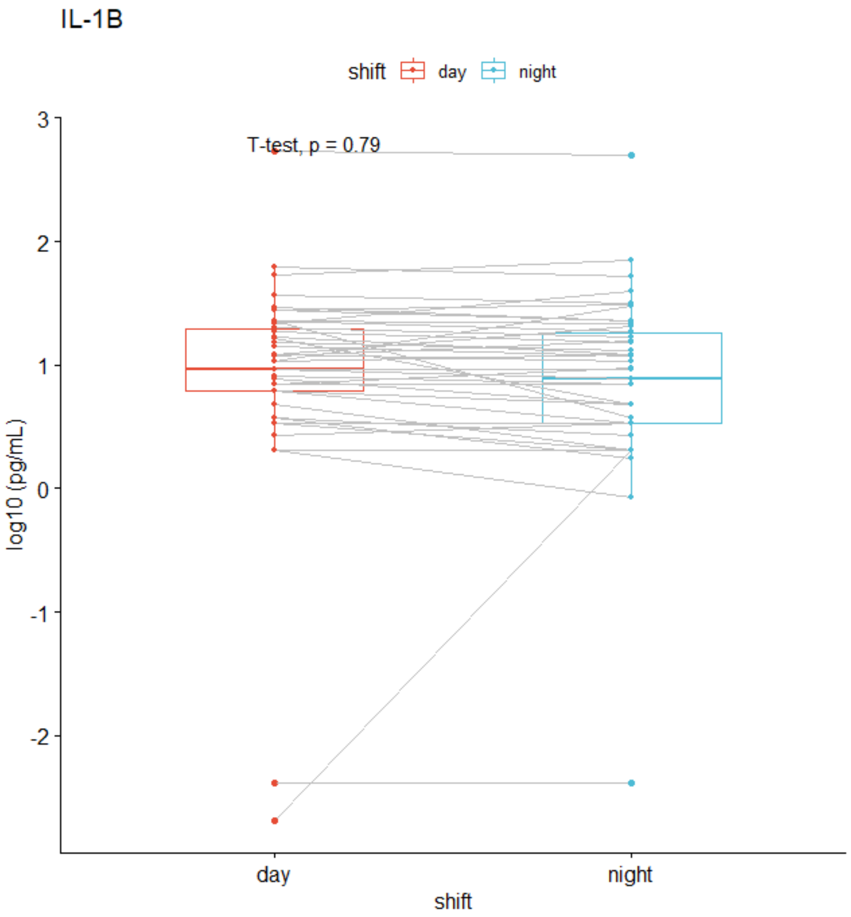

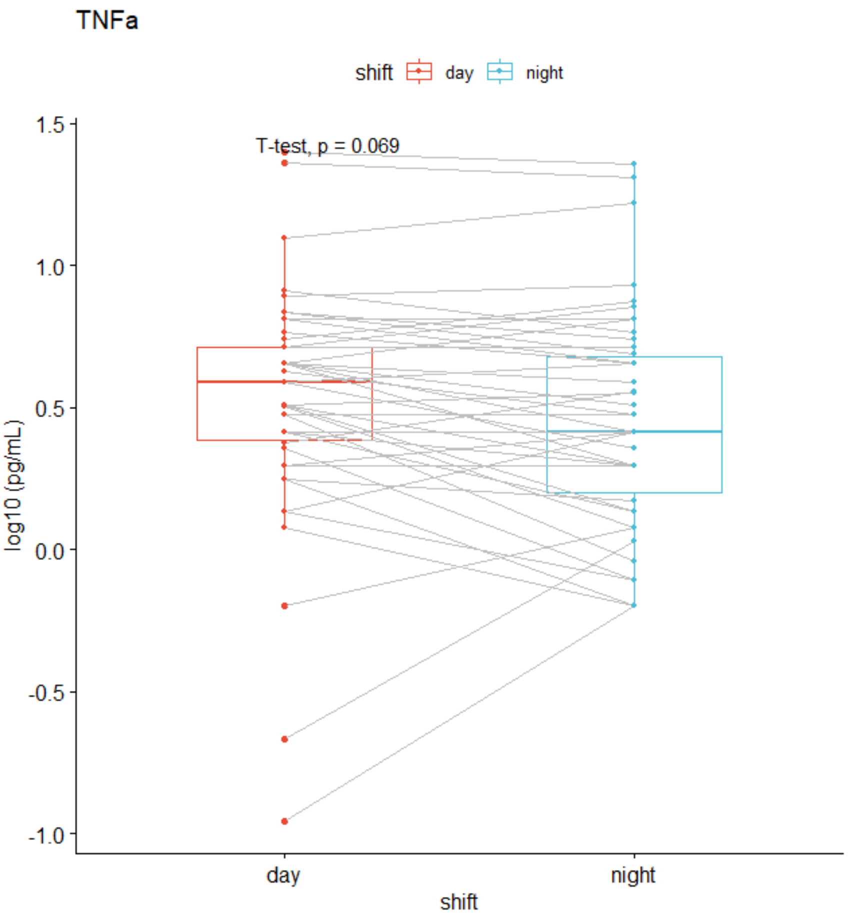

TNFa
shift
day
night
T-test, p = 0.069
log10 (pg/mL)
1.5
1.0
0.5
0.0
-0.5
-1.0
day
night
shift

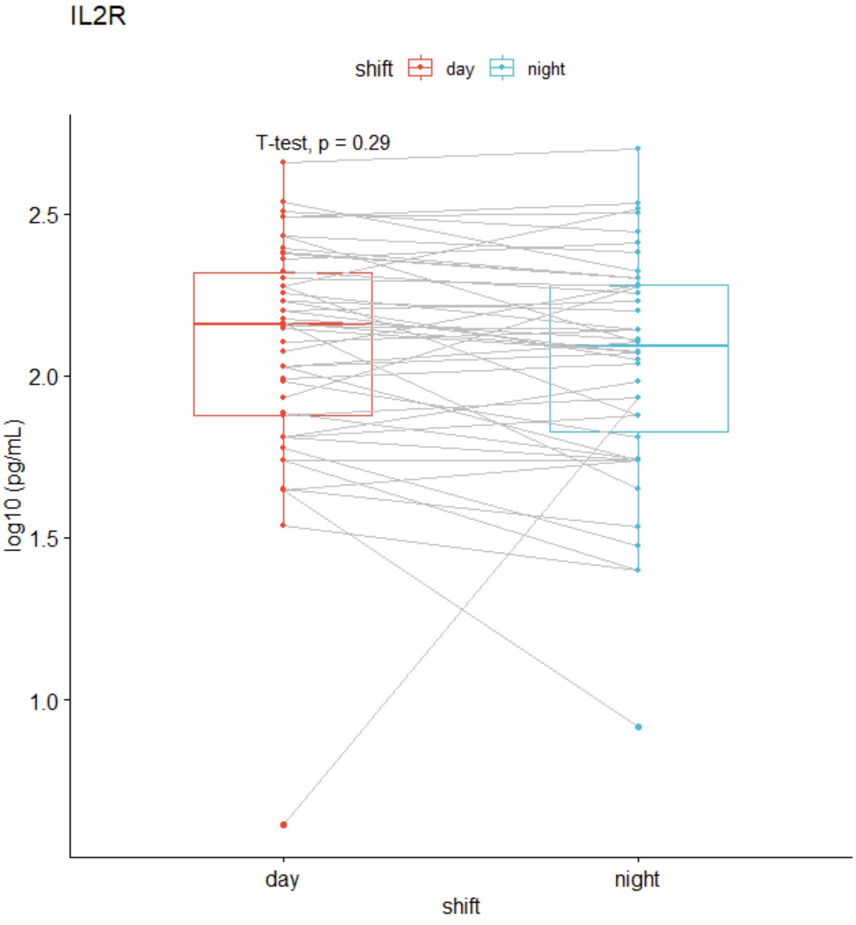

IL2R
shift
day
night
T-test, p = 0.29
log10 (pg/mL)
2.5
2.0
1.5
1.0
day
night
shift

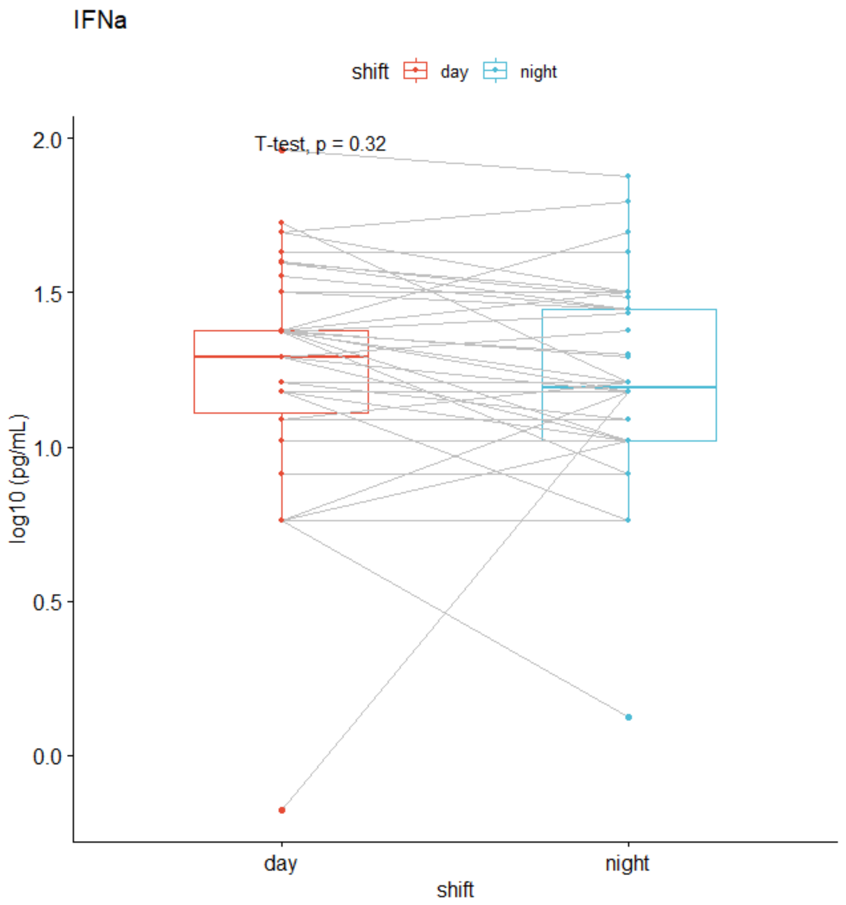
IFNa
shift day night
T-test, p = 0.32
2.0
1.5
1.0
0.5
0.0
log10 (pg/mL)
day
night
shift

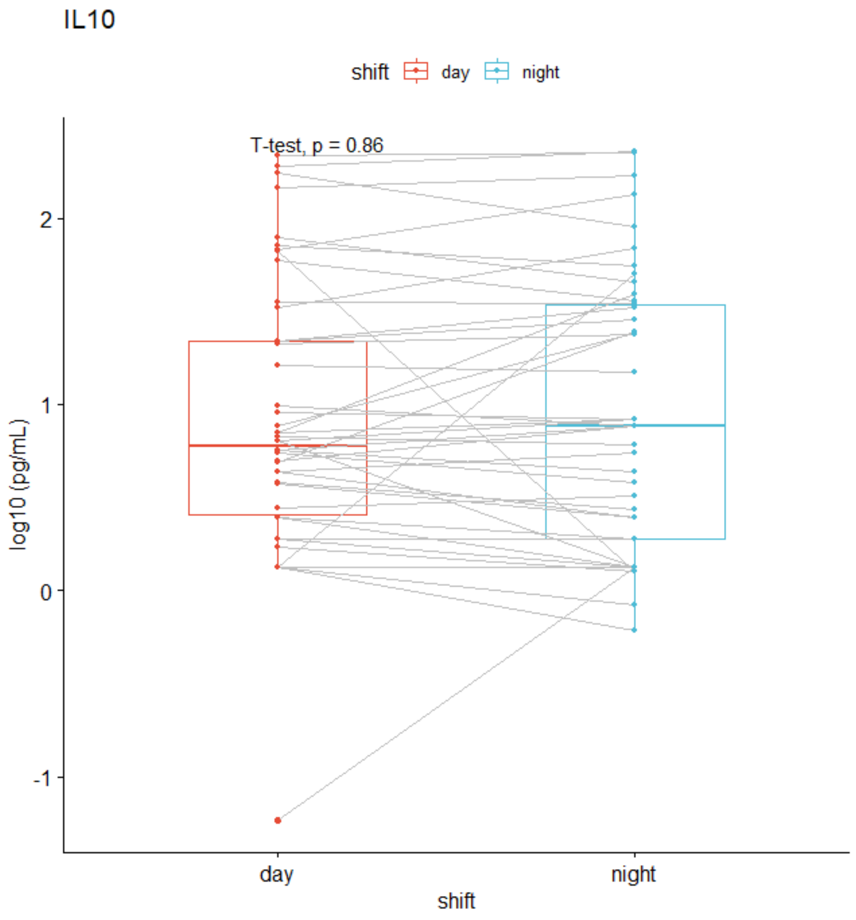
IL10
shift day night
T-test, p = 0.86
2
1
0
-1
log10 (pg/mL)
day
night
shift

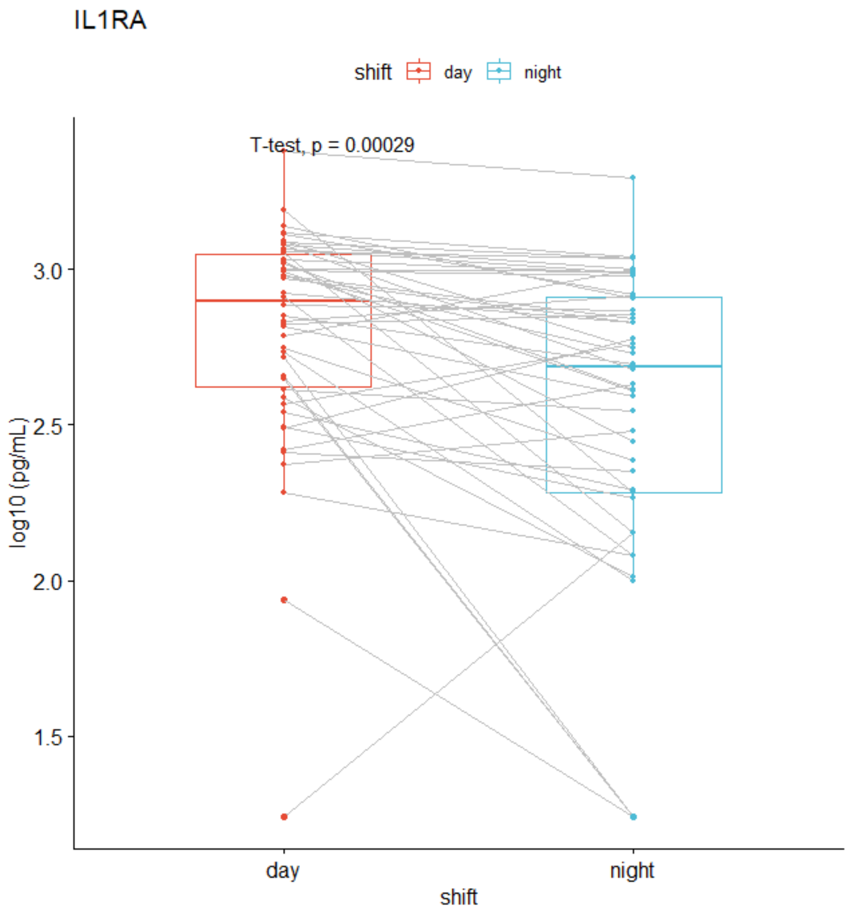
IL1RA
shift
day
night
T-test, p = 0.00029
log10 (pg/mL)
3.0
2.5
2.0
1.5
day
night
shift

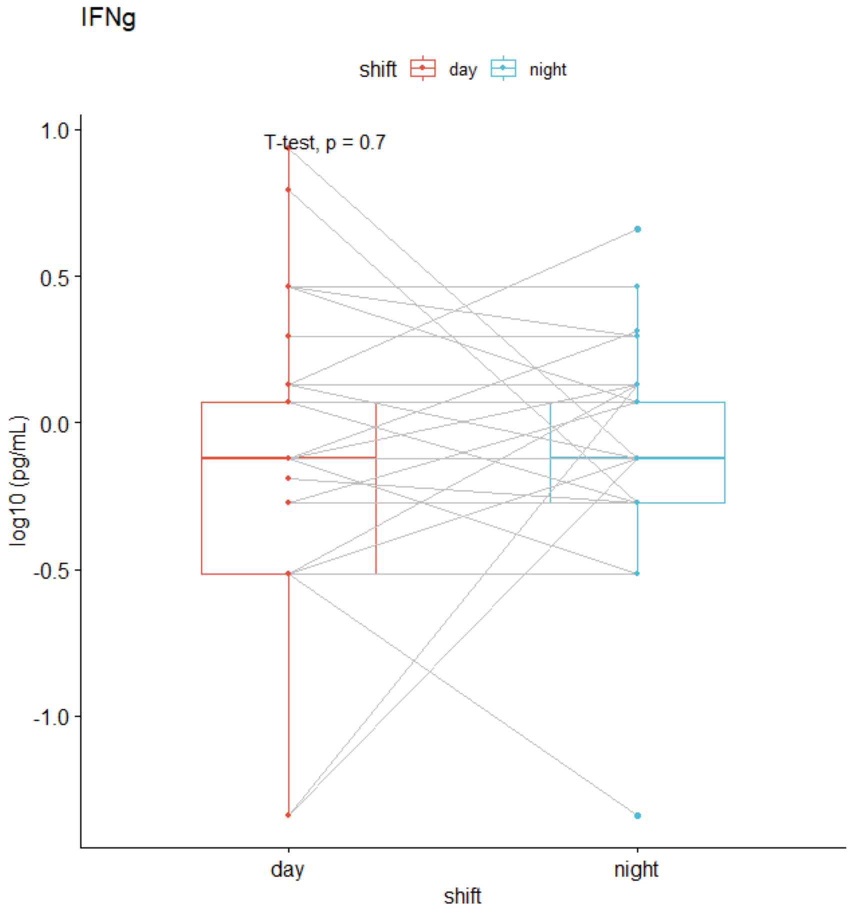
IFNg
shift
day
night
T-test, p = 0.7
log10 (pg/mL)
1.0
0.5
0.0
-0.5
-1.0
day
night
shift

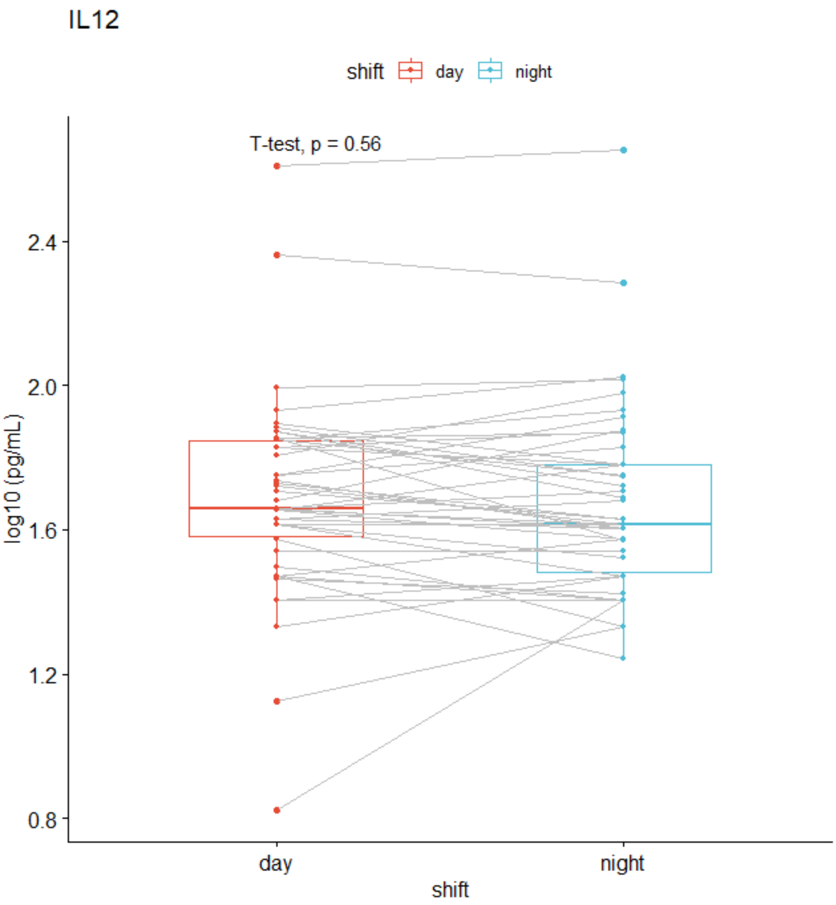
IL12
shift
day
night
T-test, p = 0.56
2.4
2.0
1.6
1.2
0.8
log10 (pg/mL)
day
night
shift

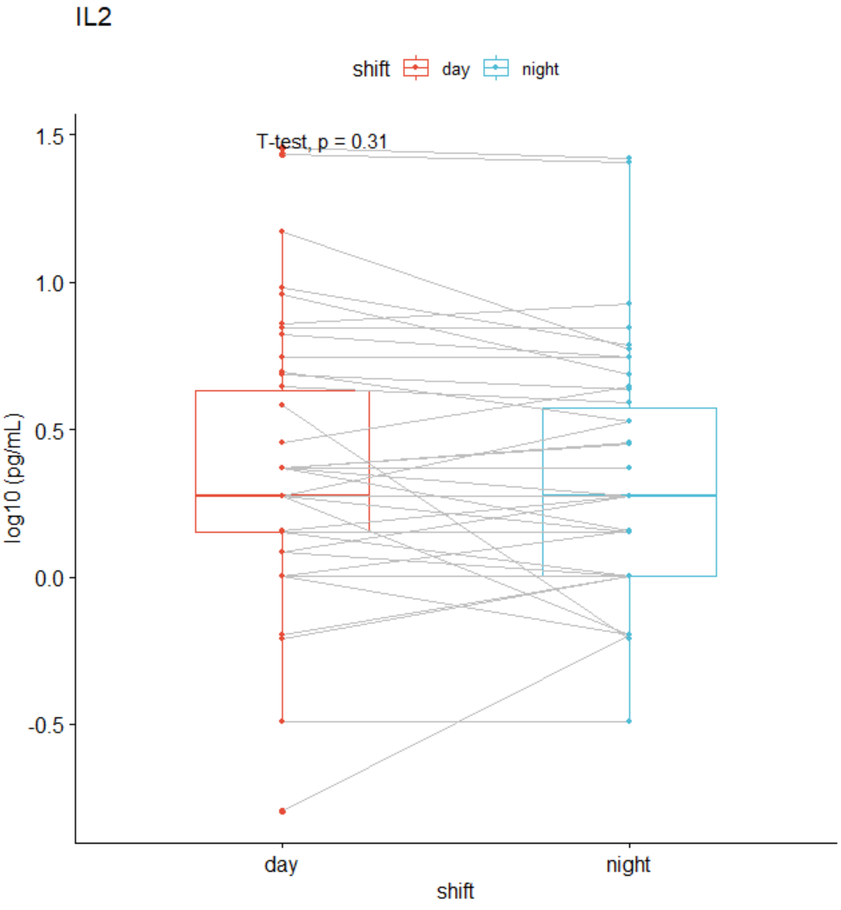
IL2
shift
day
night
T-test, p = 0.31
1.5
1.0
0.5
0.0
-0.5
log10 (pg/mL)
day
night
shift

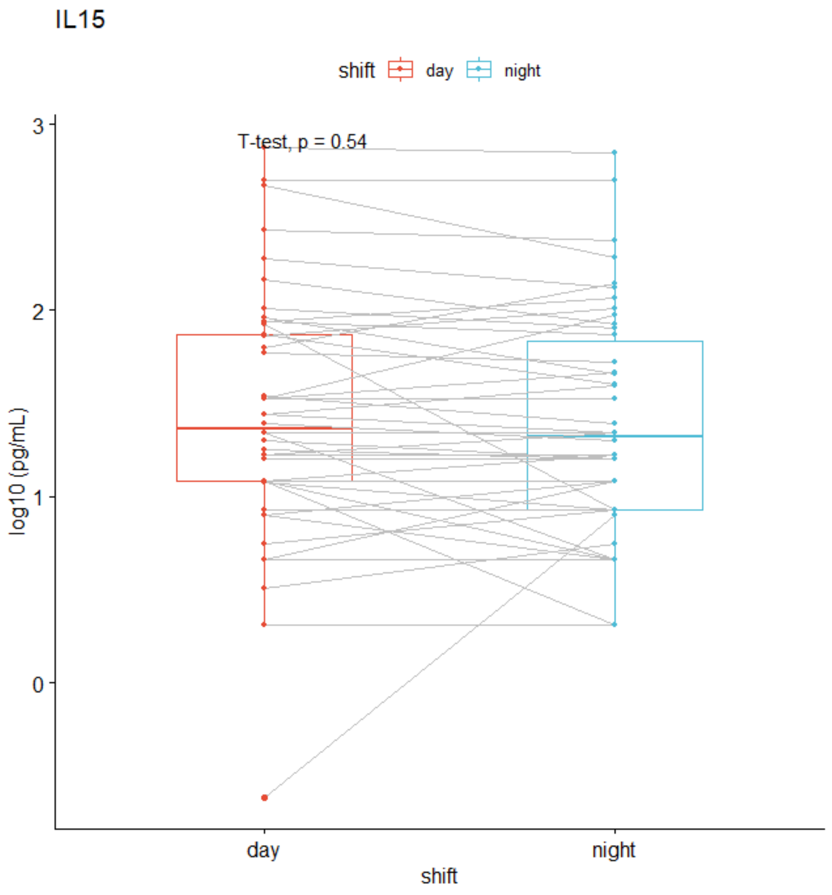
IL15
shift
day
night
T-test, p = 0.54
log10 (pg/mL)
3
2
1
0
day
night
shift

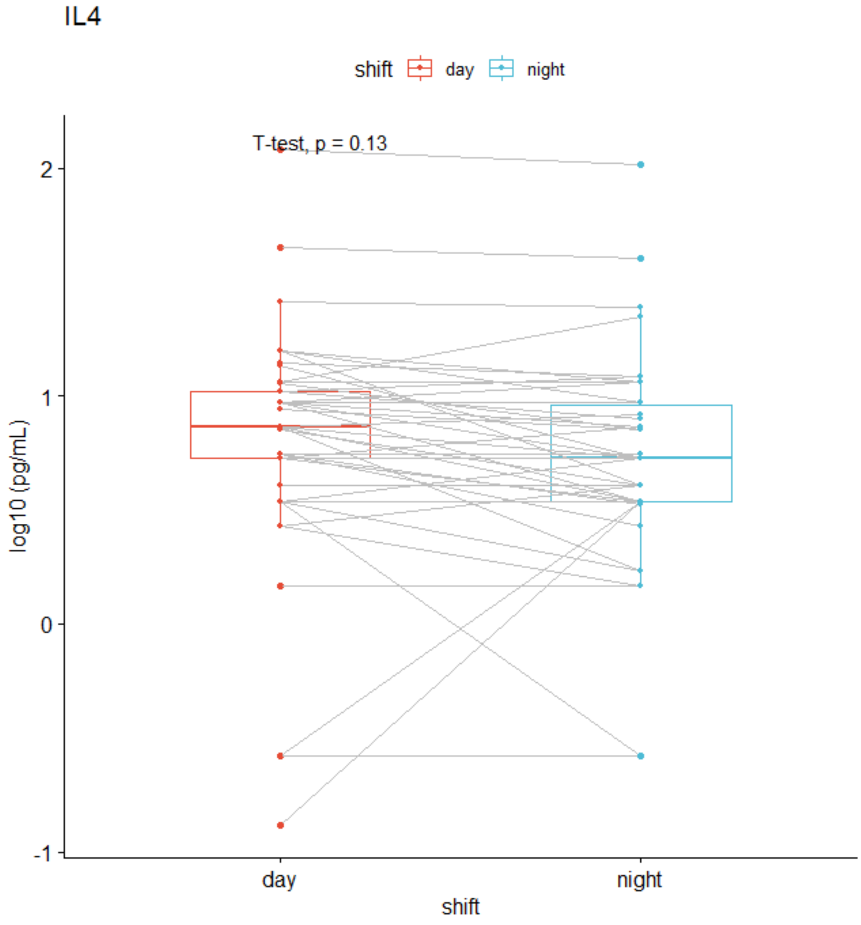
IL4
shift
day
night
T-test, p = 0.13
log10 (pg/mL)
2
1
0
-1
day
night
shift

IL5

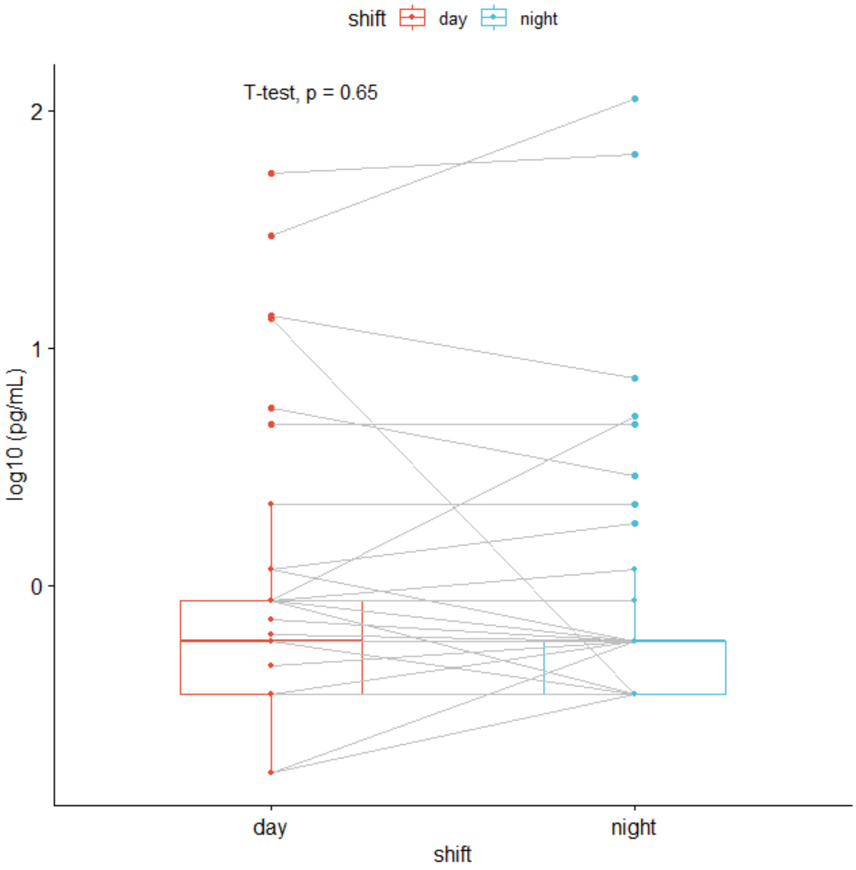
shift day night
T-test, p = 0.65
2
1
0
log10 (pg/mL)
day
night
shift

IL6

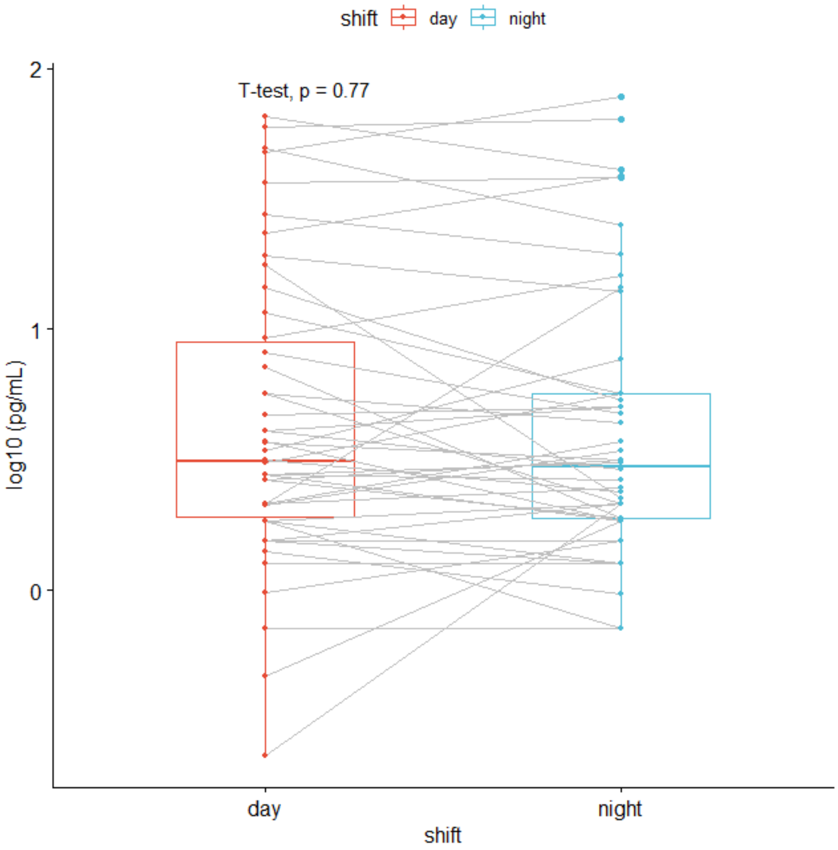
shift day night
T-test, p = 0.77
2
1
0
log10 (pg/mL)
day
night
shift

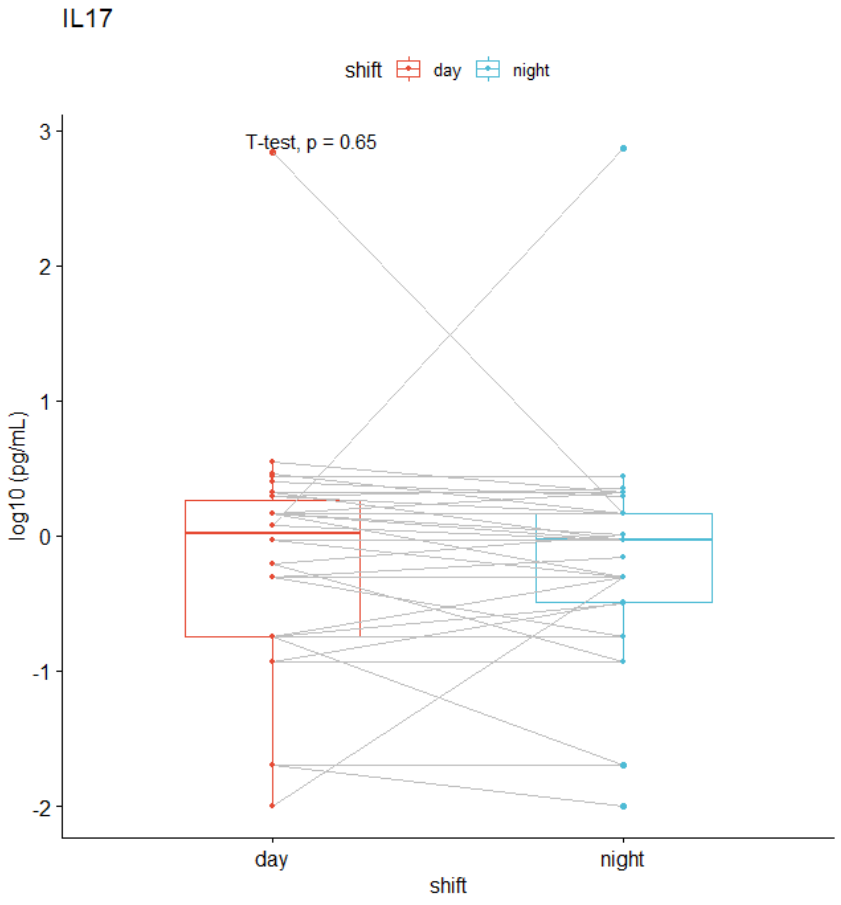
IL17
shift day night
T-test, p = 0.65
log10 (pg/mL)
3
2
1
0
-1
-2
day
night
shift

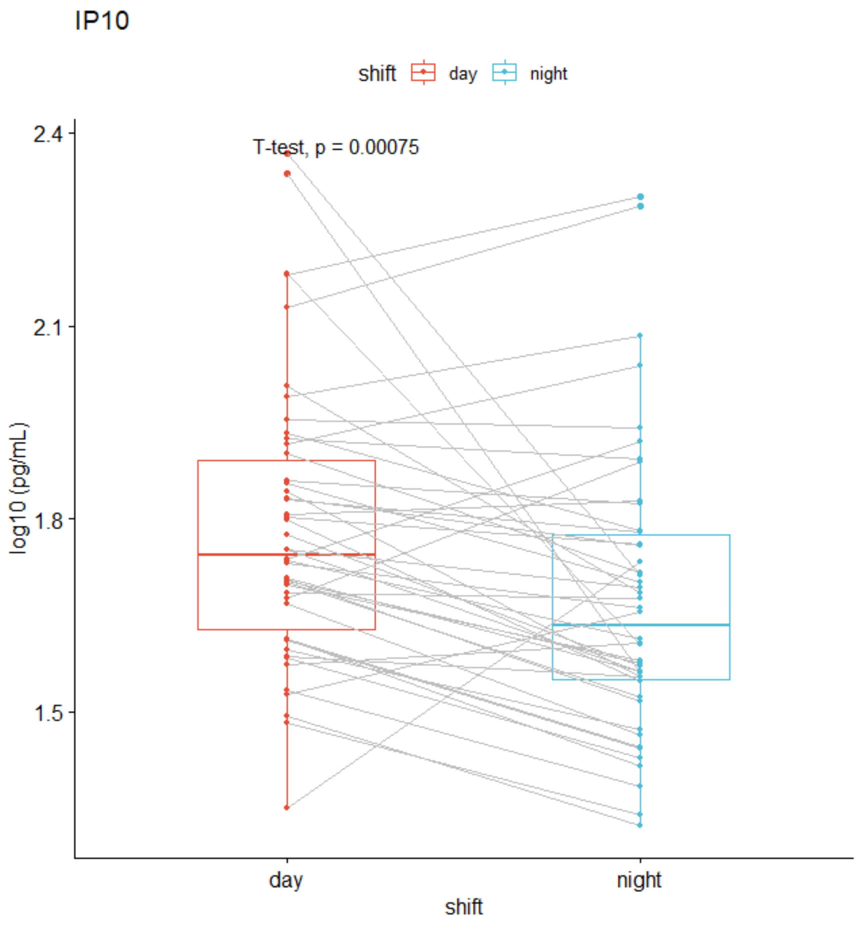
IP10
shift day night
T-test, p = 0.00075
log10 (pg/mL)
2.4
2.1
1.8
1.5
day
night
shift

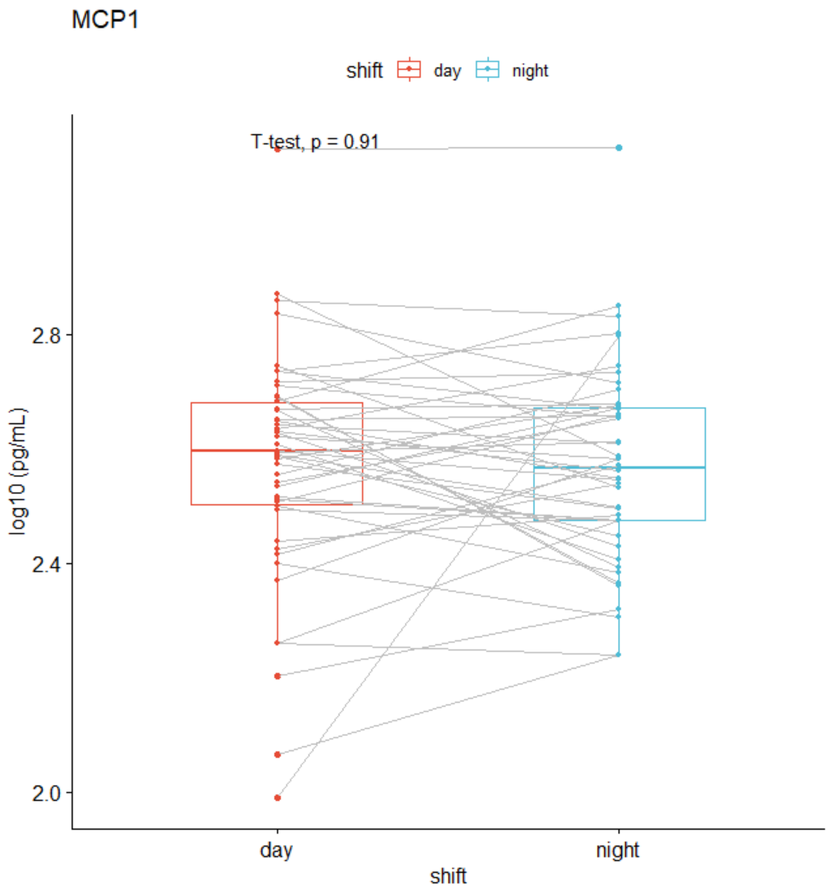
MCP1
shift
day
night
T-test, p = 0.91
2.8
2.4
2.0
log10 (pg/mL)
day
night
shift

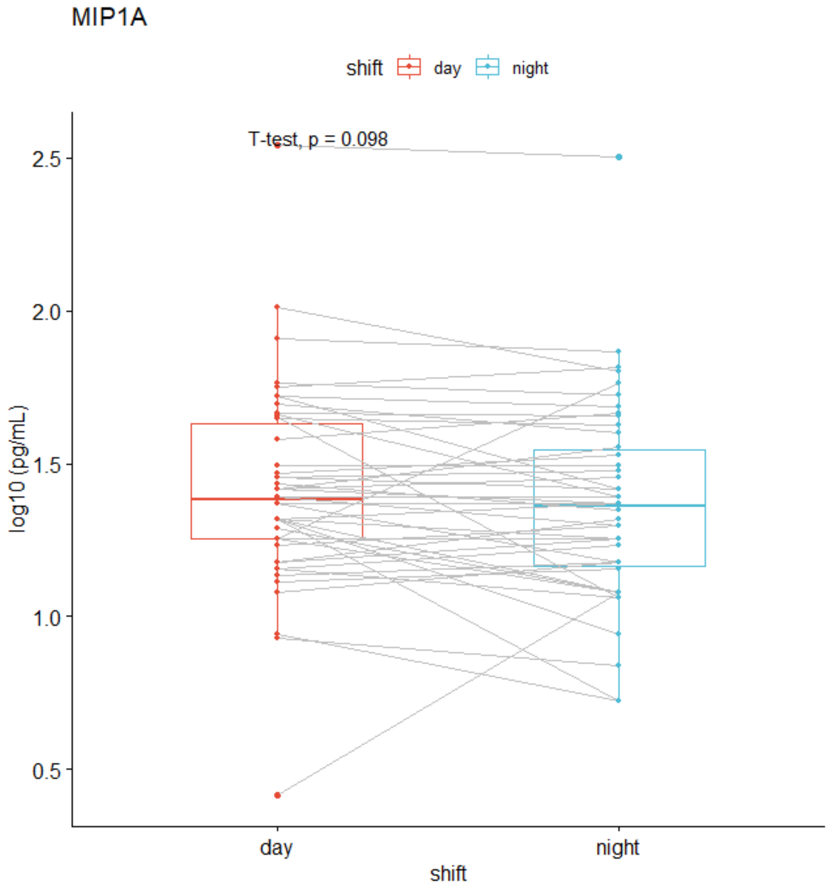
MIP1A
shift
day
night
T-test, p = 0.098
2.5
2.0
1.5
1.0
0.5
log10 (pg/mL)
day
night
shift

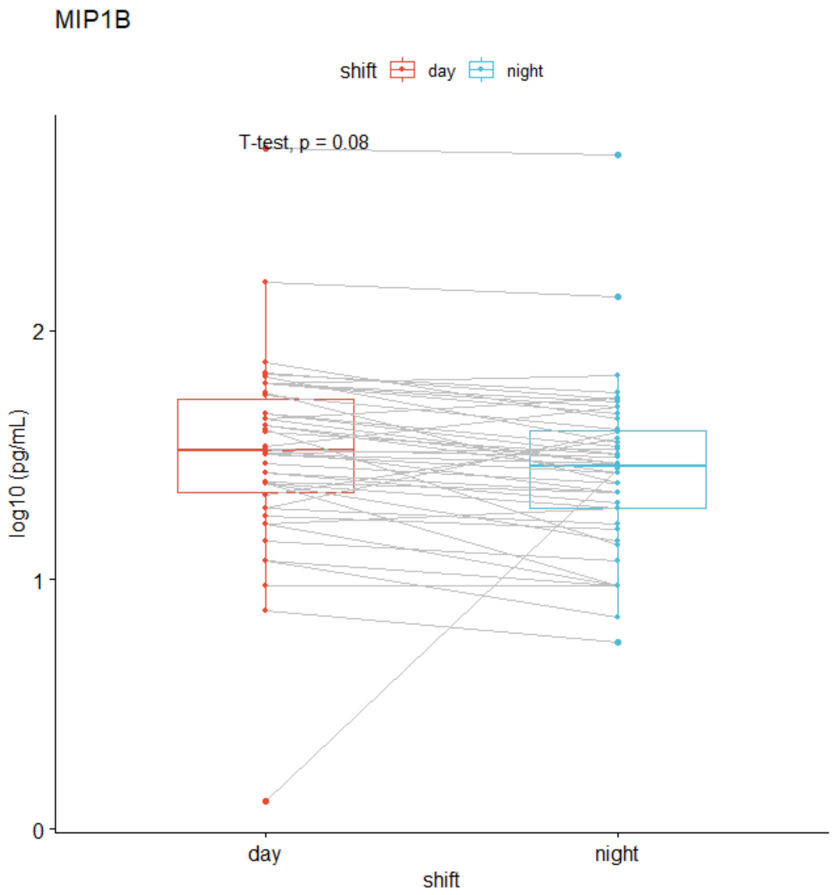
MIP1B
shift
day
night
T-test, p = 0.08
log10 (pg/mL)
0
1
2
day
night
shift

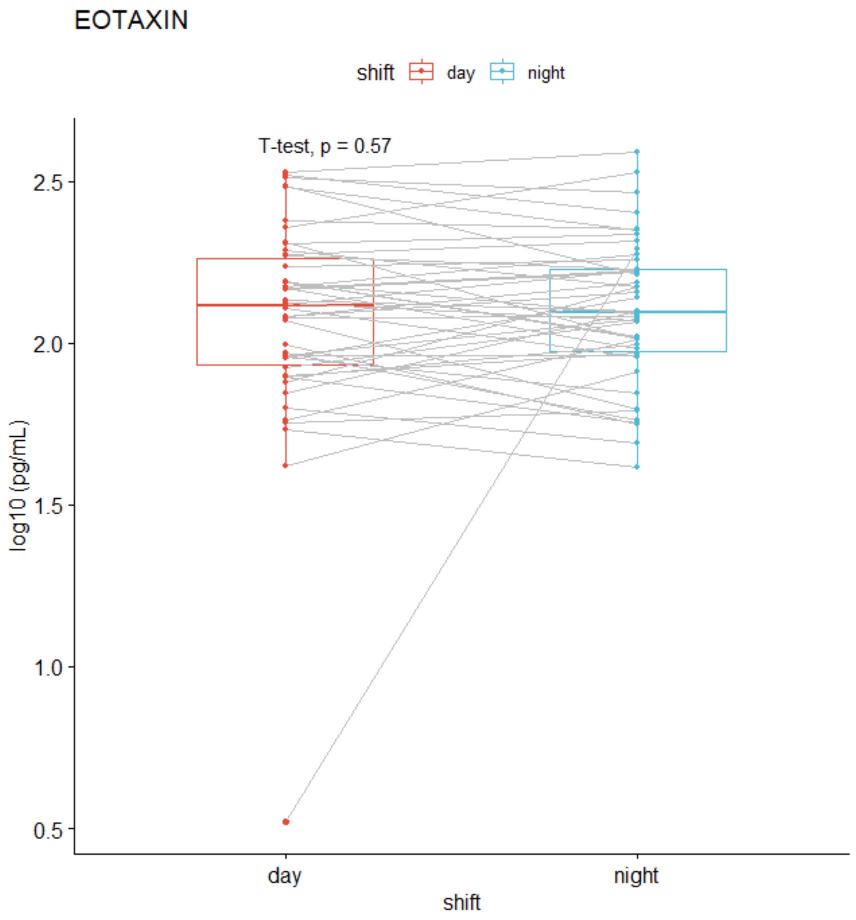
EOTAXIN
shift
day
night
T-test, p = 0.57
log10 (pg/mL)
0.5
1.0
1.5
2.0
2.5
day
night
shift

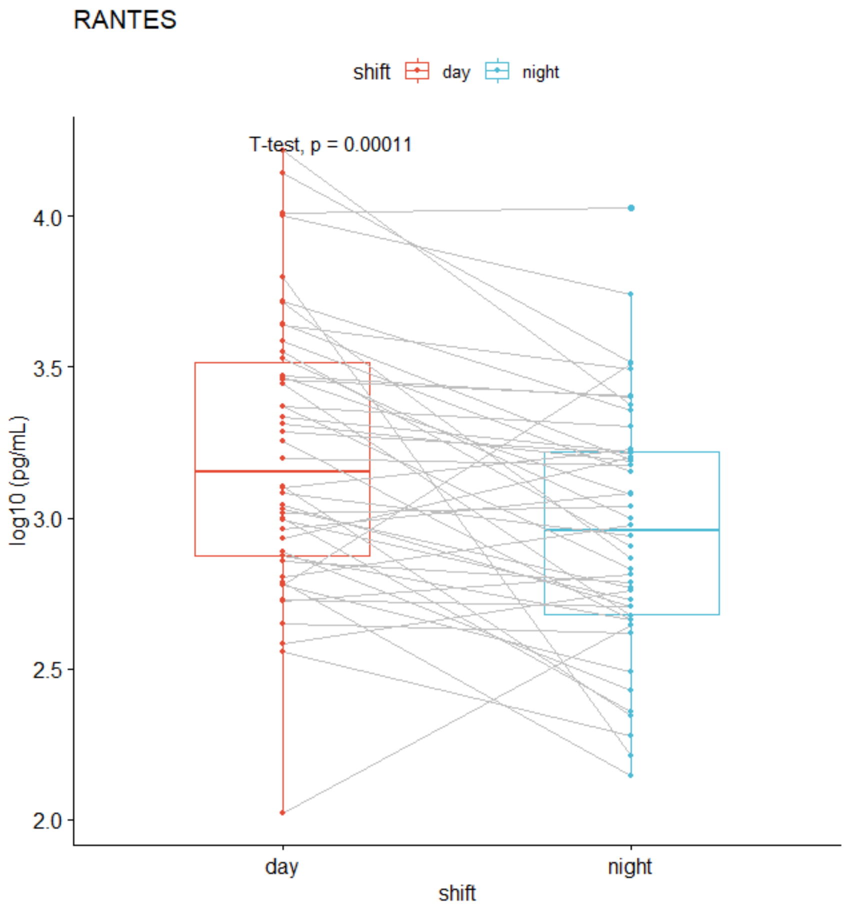
RANTES
shift
day
night
T-test, p = 0.00011
log10 (pg/mL)
4.0
3.5
3.0
2.5
2.0
day
night
shift

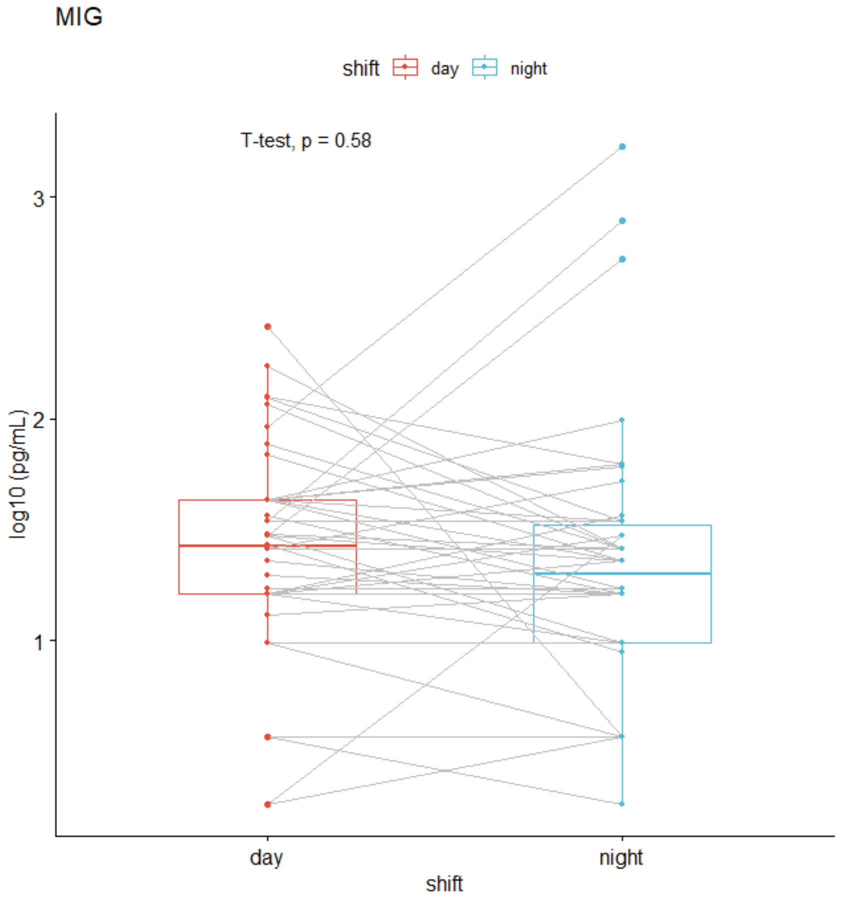
MIG
shift
day
night
T-test, p = 0.58
log10 (pg/mL)
3
2
1
day
night
shift

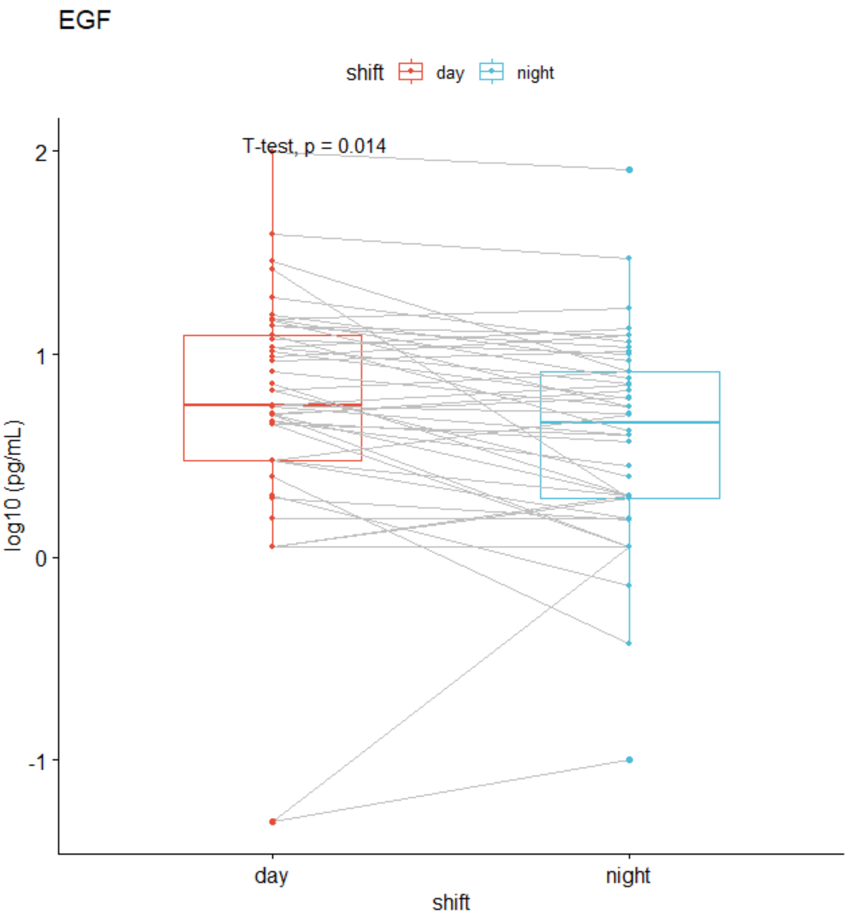
EGF
shift day night
T-test, p = 0.014
2
1
0
-1
log10 (pg/mL)
day
night
shift

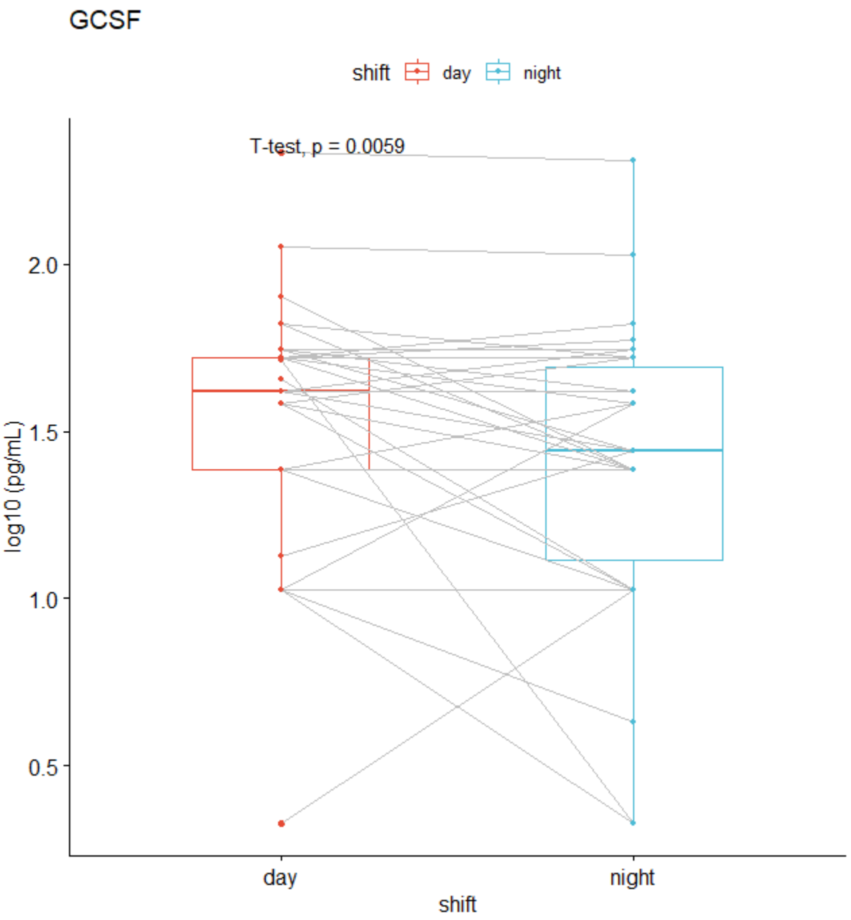
GCSF
shift day night
T-test, p = 0.0059
2.0
1.5
1.0
0.5
log10 (pg/mL)
day
night
shift

GMCSF

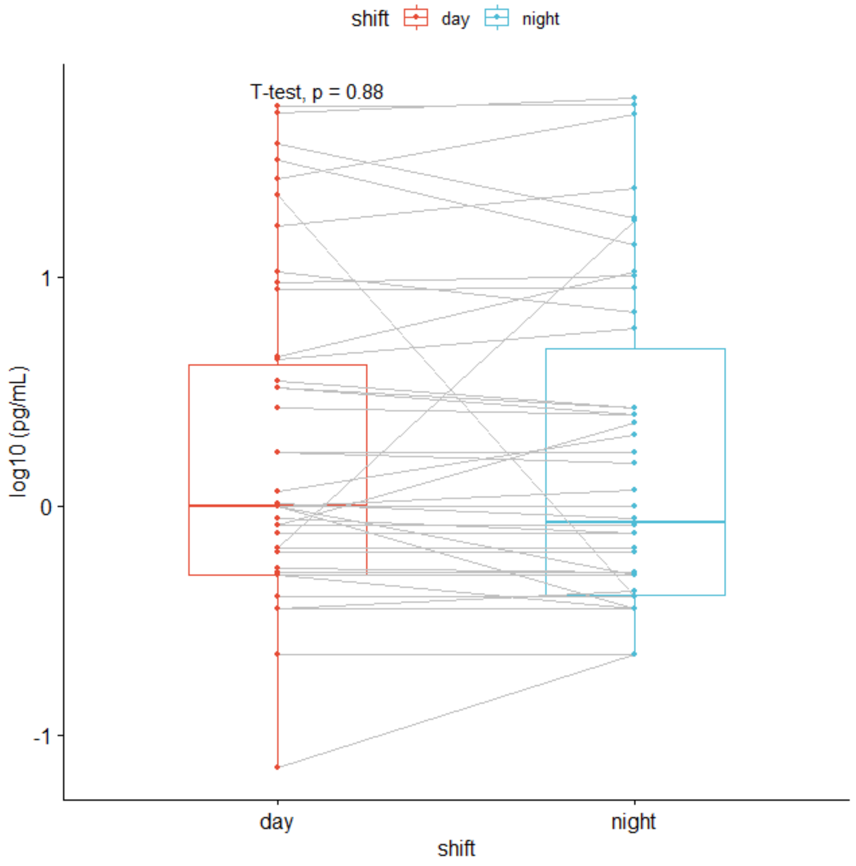

shift
day
night
T-test, p = 0.88
log10 (pg/mL)
1
0
-1
day
night
shift

HGF

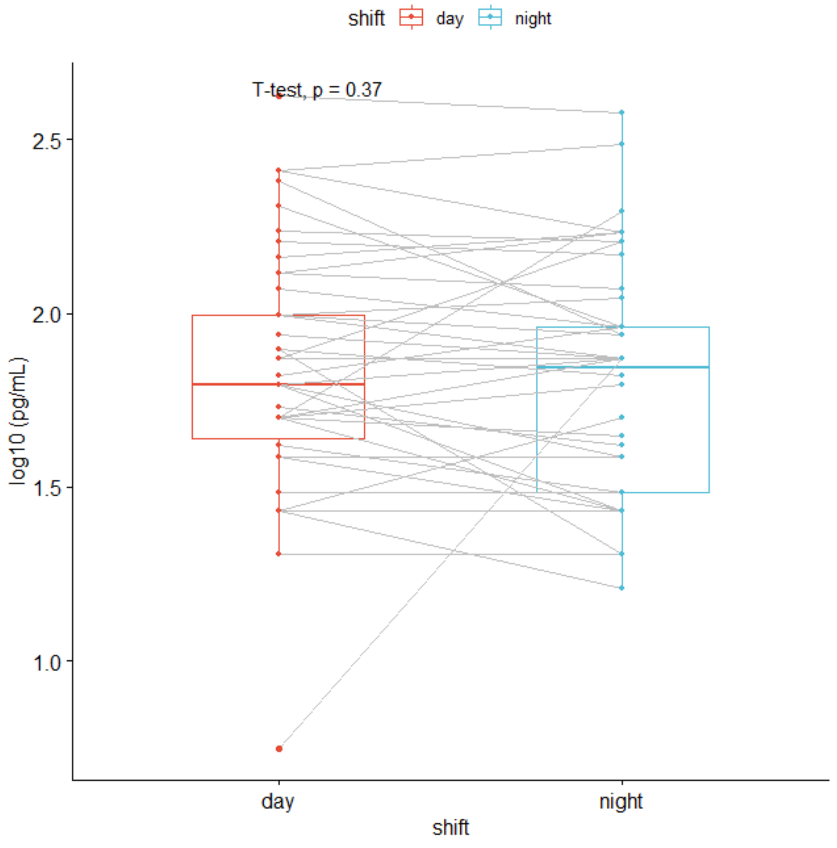

shift
day
night
T-test, p = 0.37
log10 (pg/mL)
2.5
2.0
1.5
1.0
day
night
shift

VEGF

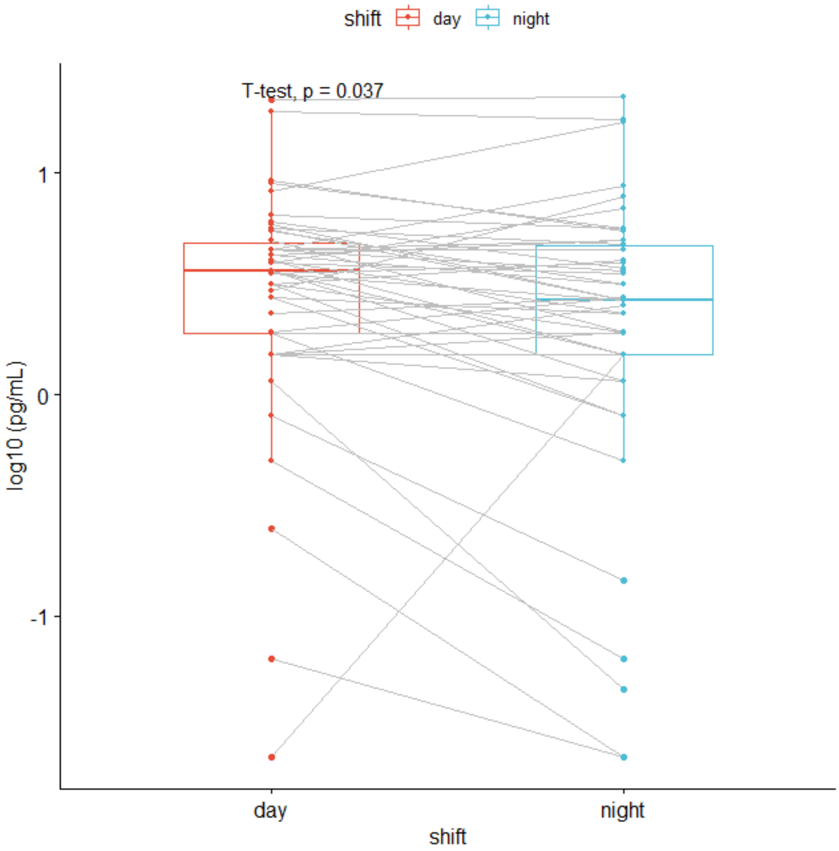
shift day night
T-test, p = 0.037
1
0
-1
log10 (pg/mL)
day
night
shift

FGF

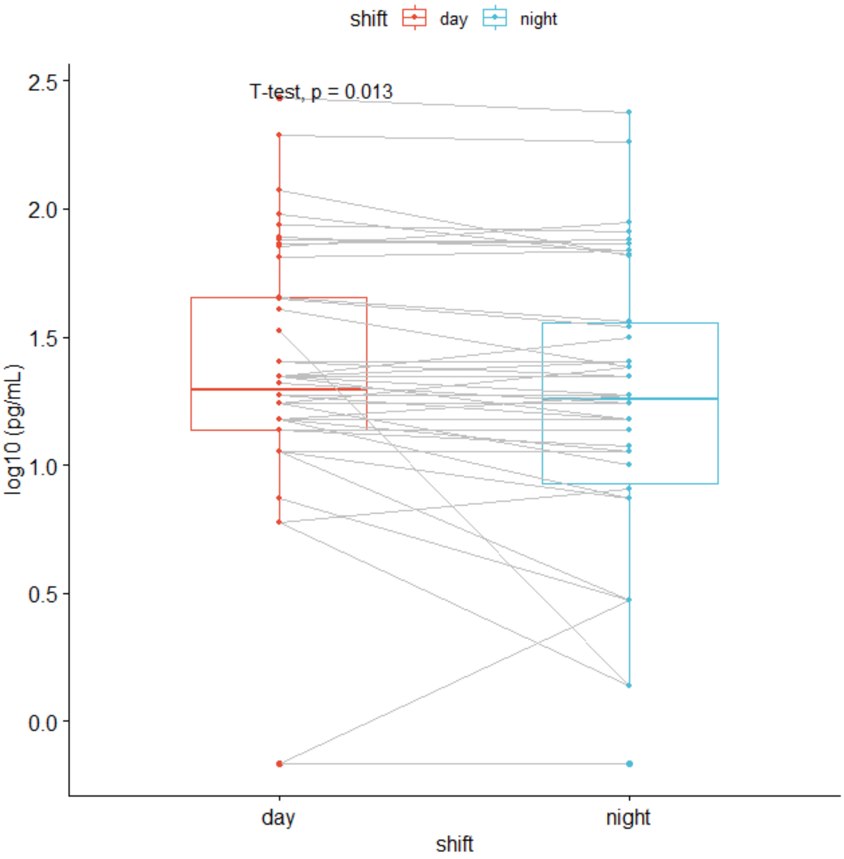
shift day night
T-test, p = 0.013
2.5
2.0
1.5
1.0
0.5
0.0
log10 (pg/mL)
day
night
shift
